# Effectiveness and Feasibility of Digital Pulmonary Rehabilitation in Patients Undergoing Lung Cancer Surgery: Systematic Review and Meta-Analysis

**DOI:** 10.2196/56795

**Published:** 2024-11-11

**Authors:** Taiping Lu, Ting Deng, Yangyang Long, Jin Li, Anmei Hu, Yufan Hu, Li Ouyang, Huiping Wang, Junliang Ma, Shaolin Chen, Jiale Hu

**Affiliations:** 1 Nursing Department Affiliated Hospital of Zunyi Medical University Zunyi China; 2 School of Nursing Zunyi Medical University Zunyi China; 3 Thoracic Surgery Department Affiliated Hospital of Zunyi Medical University Zunyi China; 4 Department of Oncology Shenzhen People's Hospital Shenzhen China; 5 School of Nursing Philippine Women's University Manila Philippines; 6 Department of Nurse Anesthesia College of Health Professions Virginia Commonwealth University Virginia American Samoa

**Keywords:** app-based, digital rehabilitation, internet-based intervention, lung cancer, perioperative pulmonary rehabilitation, systematic review, telerehabilitation

## Abstract

**Background:**

Pulmonary rehabilitation (PR) has been shown to effectively support postsurgical recovery in patients with lung cancer (LC) at various stages. While digital PR programs offer a potential solution to traditional challenges, such as time and space constraints, their efficacy and feasibility for patients undergoing LC surgery remain unclear.

**Objective:**

This systematic review aims to assess the feasibility and effectiveness of digital PR programs for individuals undergoing LC surgery.

**Methods:**

A systematic review was conducted, retrieving data from 6 English and 4 Chinese databases from their inception to January 1, 2024. References in related studies were also manually reviewed. The primary outcomes assessed were physical capacity, lung function, and the incidence of postoperative pulmonary complications (PPCs). The secondary outcomes were compliance, hospital stay, chest tube duration, anxiety, depression, and quality of life. Where applicable, recruitment and withdrawal rates were also evaluated. Meta-analysis and descriptive analysis were used to assess the outcomes.

**Results:**

A total of 5 randomized controlled trials and 6 quasi-experimental studies (n=1063) were included, with 4 studies being included in the meta-analyses. Our meta-analyses revealed that digital PR reduced the decline in 6-minute walk distance (6-MWD) by an average of 15 m compared with routine PR programs from admission to discharge, demonstrating a clinically significant improvement in physical capacity (mean difference –15.00, 95% CI –25.65 to –4.34, *P*=.006). Additionally, digital PR was associated with a reduction (26/58, 45%) in the likelihood of PPCs (risk ratio 0.45, 95% CI 0.30-0.66, *P*<.001) and a reduction of 1.53 days in chest tube duration (mean difference –1.53, 95% CI –2.95 to –0.12, *P*=.03), without a statistically significant effect on postoperative hospital stay (mean difference –1.42, 95% CI –3.45 to 0.62, *P*=.17). Descriptive analyses suggested that digital PR has the potential to improve knowledge, lung function, quality of life, and self-efficacy, while reducing depression and anxiety. Notably, digital PR was found to be a safe, feasible, and acceptable supplementary intervention. Despite challenges with low recruitment, digital PR enhanced exercise compliance, increased patient satisfaction, and lowered dropout rates.

**Conclusions:**

This systematic review is the first comprehensive analysis to suggest that digital PR is a safe, feasible, acceptable, and effective intervention for promoting recovery in patients with LC after surgery. Digital PR has the potential to be a valuable supplement, expanding access to traditional PR programs. Future research should prioritize the development of interactive and inclusive digital solutions tailored to diverse age groups and educational backgrounds. Rigorous studies, including large-scale, high-quality randomized controlled trials with detailed protocols and robust methodologies, are needed to assess the short-, medium-, and long-term efficacy of digital PR, ensuring reproducibility in future research.

**Trial Registration:**

PROSPERO CRD42023430271; https://www.crd.york.ac.uk/prospero/display_record.php?RecordID=430271

## Introduction

### Background

Lung cancer (LC) is the most prevalent cancer worldwide and the leading cause of cancer-related mortality [1]. The importance of perioperative pulmonary rehabilitation (PR) for patients undergoing LC surgery cannot be overstated. Evidence demonstrates its effectiveness in significantly reducing the incidence of postoperative pulmonary complications (PPCs) [2]; improving exercise capacity, pulmonary function, and overall quality of life after lung resection [3]; and enhancing self-management [4]. However, the current implementation of perioperative PR remains suboptimal [5,6]. A key challenge related to traditional PR programs is their reliance on face-to-face education. This approach is hindered by a shortage of well-trained health care professionals, limited time and resources for education, insufficient support services for patients with LC [7,8], and difficulties in providing adequate supervision [9,10]. Notably, patients in rural and remote areas have limited access to rehabilitation teams after discharge [10], highlighting the critical need for innovative approaches to enhance the effectiveness and accessibility of PR for patients undergoing LC surgery.

The rapid popularization of the internet and mobile apps has made tele–health care easily accessible, providing patients with convenient, flexible, and extended access to evidence-based interventions for self-management, surveillance, and supportive care, especially during the pandemic [6]. Although the terms “telehealth” and “digital health” share common features, telehealth often refers more narrowly to synchronous interactions between health care providers and patients, while digital health encompasses a broader range of digital technologies in health care [11]. In this review, the term “digital PR,” also referred to as “internet-based PR,” is defined as the utilization of digital technologies or devices in PR. This includes the Internet of Things, computing platforms, connectivity, software or apps, remote monitoring, wearable devices, virtual reality, augmented reality, and sensors for health care purposes, aligning with the World Health Organization’s definition [12]. Health care providers communicate with patients through various methods, including videoconferencing, video communication systems, WeChat, and Facebook, extending beyond traditional telephone and SMS text message interactions [13].

Compared with traditional PR, digital PR offers significant advantages, including secure remote storage and transmission of tailored PR educational materials, real-time monitoring of vital parameters and health care data, guidance for patient PR practices, and improved adherence to PR programs, all unhampered by temporal or geographical constraints. This approach reduces time and costs while enhancing patients’ access to PR programs, ensuring convenience, flexibility, and extended reach [14]. Researchers have explored the efficacy of various internet-based PR programs in patients with preoperative or postoperative LC [15,16]. However, digital rehabilitation also has some disadvantages, including technical issues [17], quality assurance concerns [18], and challenges related to impersonality [19]. Furthermore, there is ongoing debate regarding the effectiveness of telerehabilitation in treating other chronic diseases [6,10]. Notably, there are no published systematic reviews that focus on the feasibility and benefits of digital PR for patients with LC before or after surgery, nor are there any meta-analysis data reported. To our knowledge, no studies have been published to address this gap.

### Objectives

This systematic review aims to explore the feasibility and effectiveness of digital PR. Additionally, we sought to determine whether it could serve as an effective alternative to replace or supplement traditional PR. Successful findings could provide valuable insights for future clinical research and practice, assist governments and policy makers in resource allocation, and promote the development of digital PR programs to improve the overall quality of health care services.

## Methods

### Design

This systematic review was registered on the PROSPERO platform (CRD42023430271), and there were no deviations from the registered protocol. The review procedures were independently conducted by 2 authors (TPL and TD), with any disagreements resolved through consensus or consultation with the third and fourth senior authors (SLC and JH).

### Ethics Considerations

As our study was a systematic evaluation, no ethical review was conducted.

### Eligibility Criteria

The inclusion criteria were as follows: (1) patients diagnosed with LC undergoing surgery across various phases (*population*); (2) at least one of the following digital technologies was used in PR: Internet of Things, computing platforms, connectivity, software or apps, remote monitoring, online video dissemination, wearable devices, virtual reality, augmented reality, and sensors for health care purposes (*interventions*); (3) the control group received conventional PR through face-to-face interactions, paper documents (such as handouts and brochures), or telephone follow-up. Quasi-experimental studies without comparisons were also considered (*comparisons*); (4) primary outcomes included physical capacity measured by the 6-minute walk distance (6-MWD) test, lung function, and the incidence of PPCs [20]. Secondary outcomes included compliance, length of hospital stay, duration of chest tube indwelling, levels of anxiety or depression, and quality of life (*outcomes*); and (5) randomized controlled trials (RCTs) and quasi-experimental studies (*study designs*).

Exclusion criteria were as follows: (1) interventions in the intervention group were delivered without the internet, solely through face-to-face interactions or telephone calls (*interventions*); (2) incomplete data or unclear outcome effects (*data*); (3) duplicate or unavailable full texts (*publications*); and (4) publications in languages other than English and Chinese (languages).

### Data Sources and Search Strategy

The search strategy was developed with the assistance of a library specialist. Databases searched included PubMed, Cochrane Library, Embase, Web of Science, MEDLINE, and CINAHL, along with Chinese databases such as CNKI, CBM, Wan Fang Database, and the China Science and Technology Journal Database (VIP Database), covering the period from inception to January 1, 2024. Detailed search strategies for each database and full search strings are provided in Multimedia Appendix 1. Additionally, references from the included studies and other relevant reviews were manually retrieved.

### Study Selection and Data Extraction

The EndNote software (Clarivate Analytics) was used for literature management and screening. After removing duplicate articles, the titles and abstracts of the remaining articles were reviewed. Subsequently, the full texts of potentially eligible studies were carefully examined based on the eligibility criteria to determine inclusion. Data extraction followed the Template for Intervention Description and Replication (TIDieR) checklist [21] and the TIDieR-telehealth framework checklist [22]. The extraction included the following elements: author, publication year and region, study design, setting, eligibility criteria, participant characteristics, sample size, intervention details for each group, follow-up information, detailed outcome measures and time points, and outcome data.

### Risk of Bias Assessment

The risk of bias in RCTs was evaluated using the revised Cochrane Risk of Bias Tool version 2.0 [23], which comprises 5 domains with responses categorized as “yes,” “probably yes,” “probably no,” “no,” or “no information.” Overall risk was classified as “low risk of bias,” “some concerns,” or “high risk of bias” [23]. For quasi-experimental studies, the Joanna Briggs Institute’s appraisal tool [24] was utilized, consisting of nine 9 questions scored as “yes” (1 point), “no” (0 points), “unclear” (0 points), or “not applicable” (0 points). Scores of less than 5 out of 9 indicated low methodological quality [24].

### Statistical Analysis

All statistical analyses were conducted using Review Manager 5.4 software (RevMan, The Cochrane Collaboration). When appropriate, mean differences and 95% CIs were reported for continuous outcomes (eg, 6-MWD), while risk ratios and 95% CIs were provided for dichotomous outcomes (eg, the incidence of PPCs). A meta-analysis was conducted only when the outcome variables being assessed were the same, the timing of outcome measurements was comparable, and the interventions were consistent across studies. If clinical heterogeneity was present in any of these conditions, a descriptive analysis was performed instead of a meta-analysis. Results were visualized using forest plots, and heterogeneity was assessed using *I*^2^ statistics. The fixed-effects model was applied for data combination when *I*^2^ was less than 50%; otherwise, the random-effects model was used for the meta-analysis [25]. Publication bias was not assessed due to the limited number of included studies.

## Results

### Selection Process

**Figure 1:**
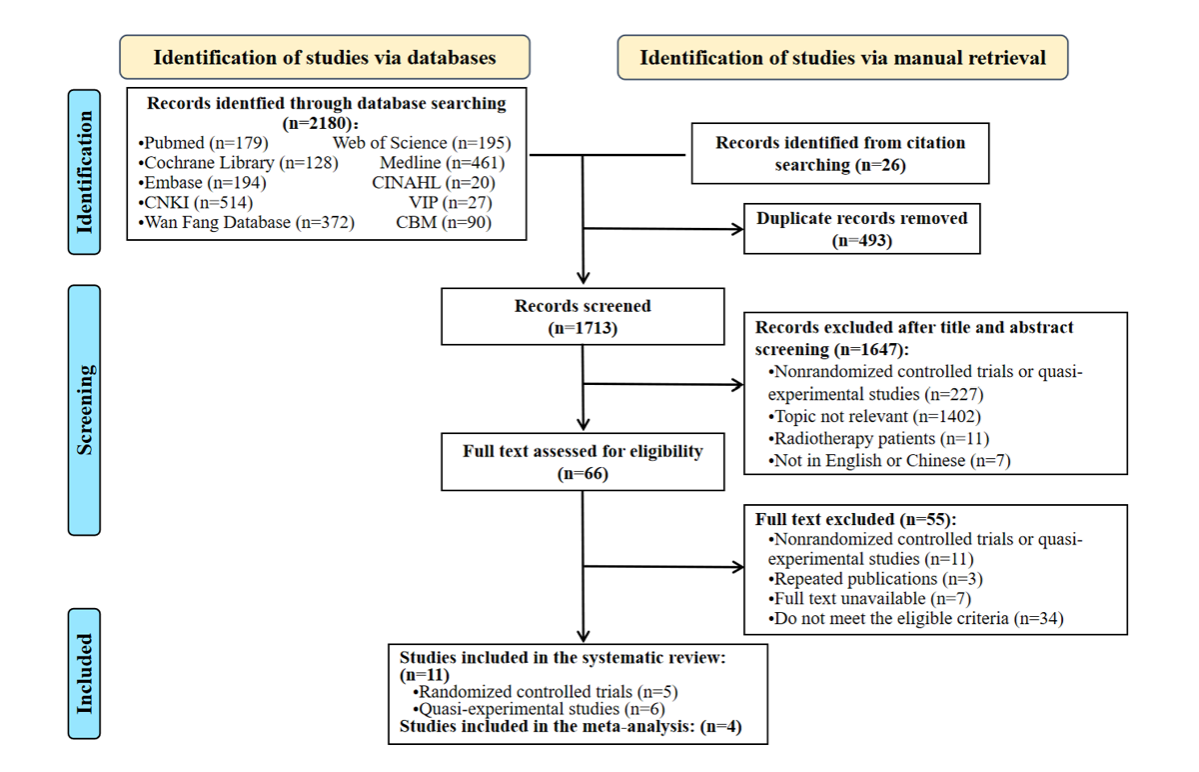
PRISMA 2020 Flow Diagram of Study Selection.

A total of 2180 articles were initially retrieved from electronic databases, with an additional 26 articles found through manual searches. After a thorough screening process, 11 trials [15,16,26-34] were included, comprising 5 RCTs [27,28,30-32] and 6 quasi-experimental studies [15,16,26,29,33,34] (2 single-arm before-after studies and 1 pilot study). Four studies [26,27,30,34] underwent meta-analysis, while descriptive analyses were conducted for the remaining 7 studies. The selection process is visually presented in the flowchart (Figure 1). Our systematic review rigorously follows the PRISMA (Preferred Reporting Items for Systematic reviews and Meta-Analyses) checklist [35], with details provided in Multimedia Appendix 2 (also see [35]).

### Characteristics of Included Studies

The detailed characteristics of the included studies are presented in Multimedia Appendix 3 (see also [15,16,26-34]): 11 studies, involving a total of 1063 patients with LC (control group: n=501; intervention group: n=562) and 22 family caregivers, were conducted across 4 countries: 6 in China, 2 in the United States, 2 in Korea, and 1 in Britain; 8 studies were published in English and 3 in Chinese. Of these, 10 studies focused solely on patients undergoing LC surgery, while 1 study included both patients undergoing LC surgery and their family caregivers. Of the 1085 participants, 595 (54.84%) were men and 425 (39.17%) were women. The gender was not reported by 65 participants. The average age of the participants ranged from 50.35 to 71.80 years, with sample sizes ranging from 18 to 104.

One study [16] implemented internet-based PR during the preoperative period, 7 studies [15,26-28,30,33,34] focused on the perioperative phases, and 3 studies [29,31,32] were conducted after surgery. The intervention strategies for both groups exhibited considerable heterogeneity among the studies. Seven studies [15,26,27,30,31,33,34] compared digital PR with face-to-face programs, 3 studies [27,30,33] utilized online video or multimedia education, and 2 studies [28,32] implemented PR using an app in both groups. Eight studies [15,16,26,28,29,31,32,34] focused on PR utilizing mobile apps, with 7 of these [15,16,26,28,29,32,34] also incorporating wearable devices. The apps included the WeChat app [26,31], Efil Breath app [32], Fit4Surgery app [15], Smart After-Care app [29], wrist-worn Garmin vívoactive fitness device app [16], and a wearable pedometer app [34]. Additionally, the wearable devices featured a pulse oximeter [15,29,32], muscle oxygen detector [28], heart rate monitor [16,28], digital sphygmomanometer [29], digital spirometer [29], and pedometer device [34]. Eight studies [15,26-31,33] integrated face-to-face PR with digital tools.

There was significant variation among the studies regarding the modality, intensity, duration, frequency, and supervision of digital PR programs. Intervention lengths ranged from during hospitalization to 18 months, with exercise sessions lasting from 3 minutes to 1 hour, occurring 2-5 days per week, and follow-up durations extending up to 48 months. The key intervention strategies for each included study are outlined in Table 1. Additionally, none of the included studies reported adherence to the TIDieR or TIDieR-telehealth checklist. The details of the intervention protocols for both groups and each study are comprehensively presented in Multimedia Appendix 4 (also see [15,16,26-34]), following the TIDieR guidelines.

**Table 1:** Key intervention strategies of the included studies.

Study	Interventions for the control group	Interventions for the intervention group	Digital types
Ji et al [32]	Fixed pulmonary rehabilitation with an app; and aerobic exercise (walking) + resistance exercises Providers: lung cancer specialists and nurses Frequency: every day Duration: No Intensity: moderate continuous Apps for a personalized mobile pulmonary rehabilitation platform (exercises, testing, and monitoring but no recording of breathing difficulty to adapt exercise level) Initiate time: NAa Length: 12 weeks Supervision: No Followed up: 12 weeks	Completed pulmonary rehabilitation with the app for the first 6 weeks; used an interactive app for the remaining 6 weeks; additionally, aerobic exercise (walking) + resistance exercises Providers: lung cancer specialists and nurses Frequency: every day Duration: No Intensity: moderate continuous Apps for a personalized mobile pulmonary rehabilitation platform (exercises, testing, monitoring, and recording the degree of breathing difficulty to adapt exercise level) Initiate time: NA Length: 12 weeks Supervision: No Followed up: 12 weeks	A personalized mobile health–based pulmonary rehabilitation platform: app and patient monitoring website; and a personalized mobile health–based pulmonary rehabilitation platform: Efil Breath app and patient monitoring website; and wearable pulse oximeter.
Sui et al [31]	Usual care + simple education + rehabilitation guidance through a simple session by face-to-face education manual Providers: physicians and nurses Frequency: NA Duration: NA Intensity: light Initiate time: after surgery Length: 12 months Supervision: No Followed up: 60 months by telephone or clinic visit	Postoperative pulmonary rehabilitation: health education (weeks 1-12, once/week); and rehabilitation exercise guidance: walking + aerobic exercise (weeks 13-52, once/week) and psychological support (12 months, once/2 weeks) Providers: trained nurses Frequency: every day Duration: NA Intensity: moderate Initiate time: after surgery Length: 12 months Supervision: daily walking supervision by WeChat once a week for 12 months Followed up: 48 months (total 60 months) by WeChat app	WeChat app (eg, video course, nurse supervision)
Chu et al [27]	Routine perioperative care Providers: NA Frequency: NA Duration: NA Intensity: NA Initiate time: after surgery Length: No Supervision: No Followed up: No	Perioperative pulmonary rehabilitation: routine care + scanning QR code on a smartphone to watch breathing exercise video + interactive video call education Providers: rehabilitation manager and respiratory specialist Frequency: once daily from 5 to 8 days before surgery and 3 times daily within 10 days after discharge Duration: 20 minutes each time Intensity: light Initiate time: 5-8 days before surgery Length: 1 month Supervision: No Followed up: No	Online video + video call
Li et al [30]	Traditional face-to-face pulmonary rehabilitation: instructions and demonstrations of pulmonary rehabilitation techniques, including diaphragmatic breathing exercises, pursed lips breathing exercises, balloon blowing, and other breathing exercise techniques Providers: research assistants Frequency: NA Duration: NA Intensity: NA Initiate time: at admission Length: during hospitalization Supervision: No Followed up: NA	Watched an animated pulmonary rehabilitation video downloaded on an iPad; the education content was similar to the control group; breathing exercises; and patients performed breathing exercises independently Providers: research assistants Frequency: twice a day Duration: 31 minutes Intensity: light Initiate time: at admission Length: during hospitalization Supervision: bedside teach-back twice a day Followed up: NA	Online video
Liu and Pan [28]	Portable wearable device with a mobile app monitoring system—preoperative: abdominal and deep breathing training + coughing + balloon blowing (every 4 hours); postoperative: isometric muscle, lower limb flexion, and extension exercise (day 1); arm raising and bed cycling (day 2); getting out of bed and shoulder and all-around exercise (day 3); and all-around exercise (day 4, 3-5 times/day, 5-8 minutes/time) and home exercise (3 times/week, 30 minutes/time) Providers: nursing staff Frequency: every day Duration: 5-30 minutes/session Intensity: light to moderate Initiate time: before surgery Length: perioperative period Supervision: wearable device Followed up: No	Perioperative pulmonary rehabilitation same as the control group + self-confidence cultivation (direct experience + alternative experience [1 time/week, l hour/time] + verbal support + emotional counseling) Providers: nursing staff Frequency: every day Duration: 5-60 minutes/session Intensity: light to moderate Initiate time: before surgery Length: perioperative period Supervision: wearable device Followed up: No	Wearable devices with muscle oxygen detector and a heart rate monitoring app
Sun et al [33]	Usual care Providers: NA Frequency: NA Duration: NA Intensity: NA Initiate time: before surgery Length: No Supervision: No Followed up: telephone follow-up 2-4 weeks after discharge	Pre- and postoperative pulmonary rehabilitation: The intervention is based on The Chronic Care Self-Management Model; traditional (information and technical skills) + self-management education; the intervention included videos, manuals, and postdischarge phone calls at home, containing different media with different learning modalities Providers: researcher Frequency: every day Duration: NA Intensity: NA Initiate time: 3-7 days before surgery Length: 3-7 days before surgery and day 7 after discharge Supervision: No Followed up: telephone follow-up 2-4 weeks after discharge	Multimedia care model (online video)
Kadiri et al [15]	Local chronic obstructive pulmonary disease rehabilitation classes; and strength and aerobic exercise for the upper and lower body Providers: the medical team Frequency: twice a week Duration: 90 min Intensity: light to moderate Initiate time: before surgery Length: 6 weeks after surgery Supervision: No Followed up: 6 weeks after surgery	The “Fit4 surgery” app is based on a home rehabilitation structured exercise program (with integrated patient and clinician biofeedback), including 10 exercises for the upper and lower body as well as aerobic and strength exercises (>3 minutes) Providers: the medical team Frequency: NA Duration: at least 3 minutes each time Intensity: moderate Initiate time: before surgery Length: 18 months Supervision: the pulse oximeter and cloud-based server Followed up: 18 months	“Fit4 surgery” app with a Bluetooth-enabled pulse oximeter and SIM card
Finley et al [16]	NA	Any moderately intense, aerobic physical activity; and surgeon-delivered exercise prescription + an activity tracker Providers: surgeon and the project coordinator Frequency: 5 days each week Duration: 30 minutes a day Intensity: moderate intense Initiate time: before surgery Length: preoperative Supervision: Garmin vívoactive heart rate device Followed up: 16 weeks after surgery	Wrist-worn Garmin vívoactive heart rate monitoring device with the app
Yang et al [29]	NA	Smart after-care app; recording of vital signs; complete a daily subjective symptom survey; utilize video clips for pulmonary rehabilitation; and patients receive personalized diet and nutrition information Providers: rehabilitation specialists Frequency: every day Duration: NA Intensity: NA Initiate time: after surgery Length: 12 weeks Supervision: self-monitoring devices including digital sphygmomanometer, finger pulse oximeter, and digital spirometer Followed up: weekly phone calls and every 6 weeks participants returned to the clinic for follow-up for a total of 3 months	Smart after-care app with self-monitoring devices
Qin et al [26]	Routine pulmonary rehabilitation health education: lectures and pulmonary rehabilitation training guideline videos played on television every day; breathing and limb exercises (2-3 times/day, 10-15 minutes/time); and advice strengthening exercises (30 minutes/time) Providers: doctors and nurses Frequency: every day Duration: 10-30 minutes Intensity: moderate to intense Initiate time: at admission Length: during hospitalization Supervision: No Followed up: No	Pulmonary rehabilitation program based on the internet: an online pulmonary rehabilitation health education course (video + documents + picture); before admission: face-to-face WeChat group education follows a daily training schedule (average 1-2 weeks before admission); during hospitalization: face-to-face education on respiratory, limbs, and resistance exercises + pulmonary rehabilitation delivered via the app; and after discharge: follow-up using the app and official account and face-to-face education Providers: doctors, head nurses, and charge nurses Frequency: 2-3 times a day Duration: 10-15 minutes Intensity: moderate Initiate time: before admission Length: perioperative period Supervision: WeChat group regular reminder + check in the training schedule every day Followed up: one-to-one telephone or home follow-up (weeks 1, 4, and 8 after discharge)	WeChat app + official account
Chen et al [34]	Routine nursing: close monitoring of the condition; routine anti-infection, fluid infusion, and nutritional support; medication guidance; and guidance in getting out of bed Providers: NA Frequency: NA Duration: NA Intensity: NA Initiate time: NA Length: NA Supervision: No Followed up: No	Pulmonary rehabilitation based on a wearable device pedometer, bed limb activities + respiratory exercise function (5 minutes/time, 3-5 times/day), walking exercises (twice a day), and inspiratory muscle strength training Providers: NA Frequency: every day Duration: 5-15 minutes of each exercise Intensity: light to moderate Initiate time: NA Length: NA Supervision: wearable device pedometer Followed up: No	Wearable device pedometer

^a^NA: not available.

### Risk of Bias Assessment

**Figure 2:**
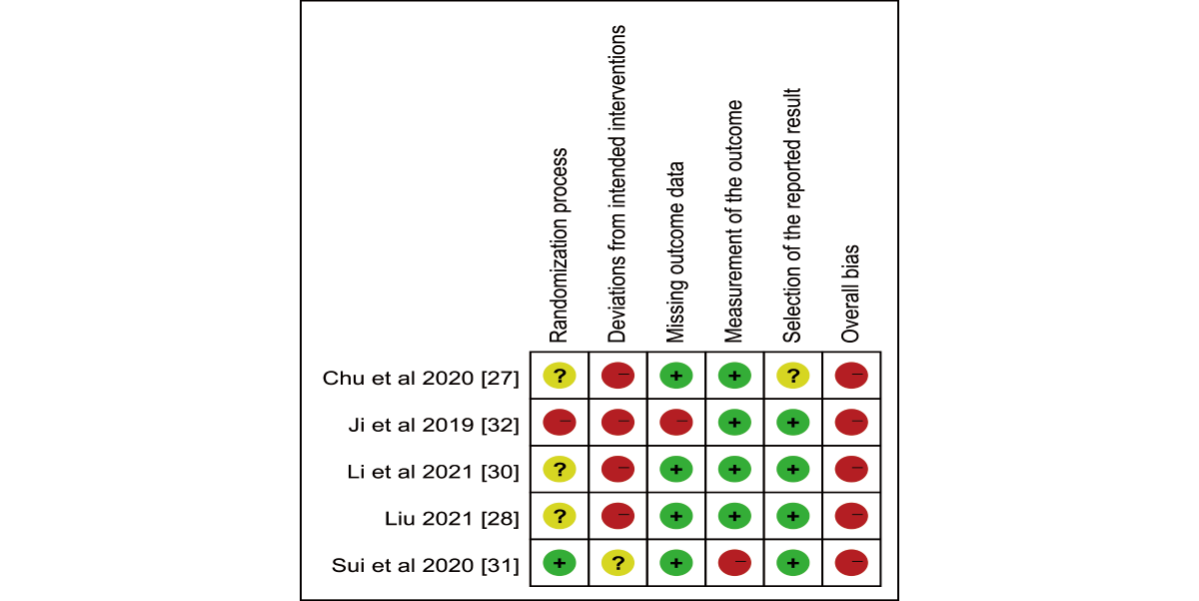
Bias risk assessment of each study.

All 5 RCTs were assessed to have an overall high risk of bias. Specifically, 1 study [32] was classified as high risk due to insufficient information on the random allocation process and concealment. Four studies [27,28,30,32] were deemed high risk because they lacked details on blinding and strategies to prevent contamination. Additionally, 1 study [32] showed a high risk of bias related to missing outcome data, while another study [31] exhibited a high risk of bias due to a lack of blinding in outcome measures. The risk of bias graph for each RCT is displayed in Figure 2 (also see [27,28,30-32]), and a summary of the risk of bias in RCTs is provided in Multimedia Appendix 5 (also see [15,16,26-34]).

Among the 6 quasi-experimental studies, 1 [15] was assessed as having an overall high risk of bias, whereas the remaining 5 studies were at low risk. Details on the risk assessment of individual quasi-experimental studies are displayed in Multimedia Appendix 5.

### Effects of the Digital PR Program

#### Physical Capacity

**Figure 3:**
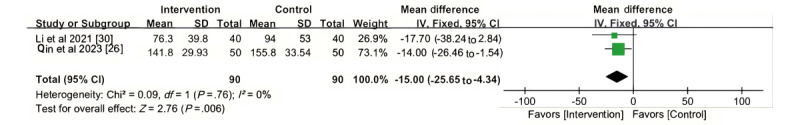
Effects of digital PR on the change in 6-WMD from admission to discharge in patients undergoing LC surgery.

Although 6 studies [16,26,28,30,32,34] reported a 6-MWD at various time points, only 2 studies [26,30] focusing on the preoperative phase were suitable for meta-analysis. The analysis revealed that the change in 6-MWD from admission to discharge in the intervention group was statistically shorter than that in the control group (Figure 3; also see [26,30]). The mean difference was 15 m, with a 95% CI ranging from 4.34 to 25.65 m (*P*=.006). One study indicated no significant difference between the 2 PR apps in postsurgery patients during unclear phases [32]. Interestingly, 2 studies [26,30] demonstrated a decrease in 6-MWD at discharge compared with admission, whereas 5 studies [16,26,28,30,34] reported a significant improvement.

Mobile app–based PR demonstrated significant improvements in 2-MWD [29], lower limb muscle strength [29], modified Medical Research Council (mMRC) scores [32], and Chronic Obstructive Pulmonary Disease Assessment Test scores [34], although there was no improvement in upper limb muscle strength [29] after the intervention.

#### Lung Function

Only 2 studies [28,34] reported lung function indicators, each using diverse metrics and reassessment days that could not be combined. Therefore, a descriptive analysis was applied. The forced vital capacity (FVC), forced expiratory volume in 1 second (FEV_1_), and the ratio of FEV_1_ to the percent predicted for FVC (FEV_1_/FVC) in the wearable devices group were statistically better than those in the routine group (*P*<.001) [34]. Additionally, PR that included an app and self-confidence cultivation significantly increased the peak expiratory flow rate compared with the app alone [28].

#### Postoperative Pulmonary Complications

**Figure 4:**
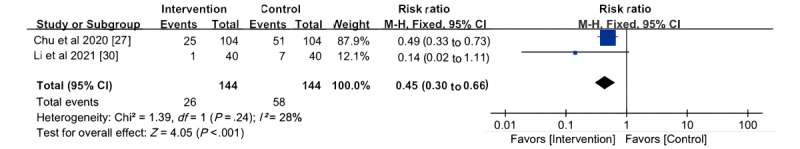
Effects of online video PR education on PPCs in patients undergoing LC surgery.

Three studies [15,27,30] reported the incidence of PPCs. One study [15] recorded a PPC rate of 9.7% following the app-based intervention. A meta-analysis of 2 studies [27,30] found that patients with LC who received online video interventions had a 45% reduced risk (risk ratio 0.45, 95% CI 0.30-0.66, *P*<.001) of experiencing PPCs compared with those receiving traditional face-to-face interventions (Figure 4; also see [27,30]).

#### Duration of Chest Tube Placement and Postoperative Hospital Stay

**Figure 5:**
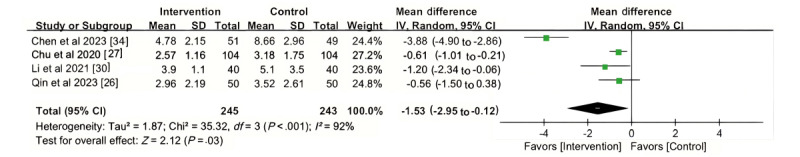
Effects of digital PR program on chest tube duration in patients undergoing LC surgery.

**Figure 6:**
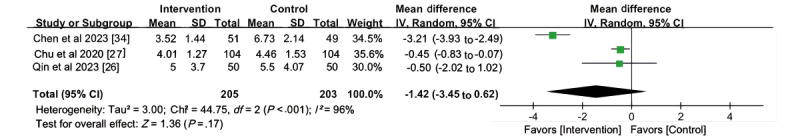
Effects of digital PR program on postoperative hospital stay in patients undergoing LC surgery.

Four studies [26,27,30,34] reported the duration of chest drainage tube indwelling, while 3 studies [26,27,34] documented the length of postoperative hospital stay. The meta-analysis revealed a statistically significant reduction in chest tube duration by 1.53 days (95% CI –0.12 to –2.95, *P*=.03) in the intervention group compared with the control group (Figure 5; also see [26,27,30,34]). However, no significant difference (95% CI –3.45 to 0.62, *P=*.17) was found between the groups regarding the length of postoperative hospital stay (Figure 6; also see [26,27,34]).

#### Quality of Life

Quality of life was assessed using various tools and time measurements in 6 studies [15,29,31-34]. Four studies [15,31,33,34] demonstrated that digital PR improved quality of life compared with the control group, including benefits for family caregivers [33]. Notably, app-based PR enhanced overall quality of life [32] and physical function, although it did not significantly improve symptoms [29] after a 12-week intervention. Furthermore, there was no significant difference (*P*=.99) between the 2 app groups [32].

#### Depression and Anxiety

Two studies [31,34] demonstrated that interventions using an app or wearable device pedometer significantly alleviated anxiety (*P*=.001) and depression (*P*=.01) compared with the control group.

#### Other Outcomes

Three studies [26,30,33] found that online video or app-based PR improved clients’ knowledge compared with routine education. Additionally, 2 studies [28,33] showed a significant improvement in self-efficacy after the intervention (*P*=.001 and *P*=.1, respectively). Moreover, 3 studies [26,29,32] indicated that app-based PR led to higher patient satisfaction, particularly with interactive apps, compared with routine education.

### Feasibility and Safety

Five studies [15,16,30,32,33] reported on the feasibility of utilizing the digital PR program. Two studies [32,33] indicated relatively low recruitment rates of 40.5% and 70%, citing difficulties in mastering the technique, as well as a lack of appropriate equipment and time. Additionally, 2 studies [16,30] reported compliance rates for the digital PR schedule at 79% and 48.3%, with higher respiratory exercise compliance observed in the animation group. Another study [33] reported high patient satisfaction along with favorable acceptability and usability ratings from both patients and family caregivers. Withdrawals were noted in all studies, with only 4 studies experiencing participant losses [15,29,31,33]. The withdrawal rate in the intervention group ranged from 10% to 32%, which was statistically lower than the control group’s withdrawal rate, which varied from 20% to 79%. No adverse events were reported across any of the studies. Detailed information is presented in Multimedia Appendix 6 (see also [15,16,26-34]).

## Discussion

### Principal Findings

To our knowledge, this work is the first systematic review exploring digital PR for patients with LC before and after surgery. The initial meta-analyses indicated that digital PR improved the 6-MWD by an average of 15 m, with a 95% CI ranging from 4.34 to 26.65 m [26,30]. This change represents a clinically significant improvement in physical capacity for patients, meeting the threshold for minimal clinically important differences. The second meta-analysis indicated that digital PR was associated with a (26/58, 45%) lower likelihood of PPCs and suggested that digital PR reduced the risk of these complications by more than half (ie, a 55% reduction) [27,30]. The final analyses identified a statistically significant reduction in chest tube duration of 1.53 days [26,27,30,34], but found no effect on postoperative hospital stay for patients undergoing LC surgery [26,27,34].

Descriptive data analyses revealed that digital PR has the potential to enhance knowledge [26,30,33], physical capacity [16,26,28-30,32,34], lung function [28,34], quality of life [15,31,33,34], and self-efficacy [28,33], while also reducing symptoms of depression and anxiety [31,34]. Furthermore, digital PR is a safe, feasible, and acceptable supplementary intervention for patients undergoing LC surgery [15,16,30,32,33]. Despite challenges with low recruitment during the enrollment phase [15,29,31,33], digital PR has been shown to improve exercise compliance [30], enhance patient satisfaction [26,29,32], and reduce dropout rates.

### Interpretation of the Findings

Our systematic review identified mobile apps as the primary intervention method, frequently combined with wearables such as pulse oximeters and heart rate monitors. However, emerging technologies such as virtual reality, augmented reality, exergame training, and intelligent robotic systems were not explored. These technologies have demonstrated potential in other rehabilitation contexts, such as improving exercise compliance for patients with chronic obstructive pulmonary disease through virtual reality–based PR [36] and reducing fall risk for older adults with exergame step training [37]. Aldebaran Robotics’ Natural Another One humanoid has successfully guided older adult patients through rehabilitation exercises, evaluating their performance for near real-time processing [38]. Future research should focus on developing integrated intelligent software and devices specifically designed for PR programs tailored to patients undergoing LC surgery. These innovations could improve education, guidance, supervision, interactivity, and cost-effectiveness, thereby optimizing the rehabilitation experience. Additionally, they may have the potential to replace traditional PR guidance in the future.

Patients undergoing LC surgery frequently experience a decline in lung function, activity intolerance, and a reduction in quality of life, with recovery taking 6-12 months. Preoperative PR is crucial for recovery; however, our review found limited research focusing on this stage, revealing only a modest improvement in walking distance. Given the short time frame and the nature of home-based preoperative care, implementing effective PR can be challenging. Mobile PR presents a promising solution, warranting further investigation through RCTs.

None of the included studies reported adherence to the TIDieR or TIDieR-telehealth checklist. Furthermore, all studies lacked critical intervention details, including information on providers, frequency, duration, intensity, initiation time, length of intervention, supervision, and follow-up methods in both the intervention and control groups. Additionally, the studies assessed various outcome variables at different time points, leading to significant clinical heterogeneity. Consequently, only 4 outcomes could be synthesized in the meta-analysis, while the remaining outcomes could only be described narratively. This variability may impact the replicability and generalizability of digital PR programs.

A total of 7 perioperative studies [15,26-28,30,33,34] and 3 post-surgical studies [29,31,32] investigated digital PR for patients with LC; however, the intervention durations and follow-up periods varied significantly. Because of the diversity in outcome indicators and assessment time points, only the 6-MWD at discharge and the incidence of PPCs could be combined for analysis, and both demonstrated significant differences. The observed improvement in physical activity aligns with findings from digital PR studies for chronic obstructive pulmonary disease [39] and chronic musculoskeletal conditions [40,41]. However, other indicators exhibited high heterogeneity, which limited the ability to conduct a descriptive analysis. To strengthen the evidence, future research should evaluate the short-, medium-, and long-term effects of digital PR.

The effectiveness of digital health systems relies on their feasibility, usability, and acceptability [42]. However, most of the included studies targeted older adult participants who were already smartphone-savvy, which limited insights into program acceptability across a broader population. Only 4 studies reported on these aspects, and none addressed the experiences of providers. Despite low recruitment rates (40%-70%) [32,33], participants in digital programs demonstrated better exercise compliance [16,30] and lower dropout rates [15,29,31,33] compared with those receiving face-to-face education. However, research indicates that telemedicine interventions do not significantly enhance patients’ adherence to exercise [39,43]. Low attendance in digital health programs among older adults is often attributed to unfamiliarity with technology [44], scarcity of devices, and time constraints [32,33]. However, once engaged, patients typically find it easy to access educational resources and supervision through apps, wearables, and the internet. Our research indicated that app-based PR programs were highly satisfying and user-friendly for both patients and caregivers. The combination of face-to-face and digital interventions in the 7 included studies leveraged the advantages of both approaches, thereby improving patient adherence. However, challenges persist in implementation, including feelings of being overwhelmed by devices, lack of internet access, or poor-quality video/audio [45]. Additionally, concerns about sensor accuracy [17] and perceptions of ineffectiveness [25,29] can hinder participation. Future programs should take providers’ experiences into account and adapt to these obstacles to enhance attendance and compliance with digital rehabilitation programs.

All 5 included RCTs were assessed to be at high risk of bias. Among the 6 quasi-experimental studies, 2 used single-arm designs, and 1 was also deemed to be at high risk of bias. This deficiency in high-quality studies may affect the overall level of evidence. Therefore, further well-designed RCTs with robust methodologies are essential to provide clearer insights into the effectiveness of digital PR in this context.

### Strengths and Limitations

Our systematic review possesses several notable strengths. First, it represents the first comprehensive analysis that highlights the benefits of digital PR for the recovery of patients undergoing LC surgery, addressing both effectiveness and feasibility. Second, we provided a detailed description of each study based on the TIDieR-telehealth framework, underscoring the necessity for future studies to enhance replicability and generalizability. Lastly, our work incorporated evidence from both English and Chinese sources, reflecting a diverse range of cultural and social contexts.

There are several limitations to consider. First, the limited number of high-quality RCTs with relatively small sample sizes may compromise the robustness of the evidence. Second, while the primary focus was on patients undergoing LC surgery, the variability in intervention initiation, follow-up durations, and chemotherapy administration across studies may have contributed to high heterogeneity. To address heterogeneity, we adhered to the Cochrane Handbook recommendations [46] and consolidated evidence from studies with similar starting phases and interventions, ensuring the rigor and reliability of our results. For instance, we summarized data for the preoperative, perioperative, and postoperative phases, as well as different follow-up time points. Meta-analysis was conducted only when the studies involved similar patient populations, interventions, and measurement time points. In instances of significant heterogeneity, we opted for descriptive analysis. Despite these efforts, considerable heterogeneity among the studies persisted. Third, the lack of standardization in reporting interventions according to the TIDieR checklist led to significant variations in study design, methodology, and outcome measures. This heterogeneity—including differences in settings, participant characteristics, intervention providers, and digital program protocols (eg, the type of digital technology and delivery; initiation timing; exercise specifics such as location, duration, frequency, intensity, supervision, guidance, modifications; and strategies to enhance fidelity)—complicates interpretation and makes it challenging to draw robust conclusions. Finally, by limiting the search to Chinese and English literature, we may have excluded valuable research published in other languages, which could further restrict the generalizability of our findings.

### Recommendations for Future Studies and Clinical Practice

First, future research should prioritize developing interactive and inclusive digital solutions that cater to diverse age groups and education levels. Second, it is essential to identify obstacles in both face-to-face and digital PR before developing any tools. Third, hybrid models that combine both methods should be explored to address challenges, enhance attendance, and ensure compliance with the PR program. Lastly, large, high-quality RCTs are needed, with a focus on clarifying blinding, allocation concealment, comparability, and specific protocol details, including duration, intensity, frequency, supervision, and adequate follow-up. Outcomes should be assessed at different time points to evaluate the short-, medium-, and long-term feasibility and effectiveness of various digital PR protocols. Key recommendations for health care practices and policies should be emphasized in future research, including the development of evidence-based digital PR guidelines to standardize the use of digital tools. Additionally, integrating digital programs into in-person rehabilitation can help address limitations of face-to-face interactions, while formulating telehealth policies will enhance patient access and options. Finally, future studies should adhere to the TIDieR [21] and TIDieR-telehealth frameworks [22], as well as relevant reporting guidelines such as CONSORT (Consolidated Standards of Reporting Trails) [47] and the Transparent Reporting of Evaluations with Nonrandomized Designs (TREND) for quasi-experimental studies [48], to ensure robust and replicable research.

### Conclusions

This systematic review highlights the safety, feasibility, and efficacy of digital PR programs for patients undergoing LC surgery. Although challenges related to recruitment and attendance persist during enrollment, these programs have demonstrated benefits in enhancing physical activity, reducing the occurrence of PPCs, and shortening the duration of chest tube placement. Furthermore, digital PR programs offer potential improvements in exercise compliance, lung function, quality of life (excluding symptoms), self-efficacy, patient satisfaction, and mental health. While rigorous research is still necessary, our findings suggest that digital PR can serve as a valuable supplement to expand access to rehabilitation. These programs provide flexible, self-directed exercise options, improve continuity through extended supervision, and enhance recovery for patients undergoing LC surgery.

## Appendix

Multimedia Appendix 1 Search strategy.

Multimedia Appendix 2 PRISMA (Preferred Reporting Items for Systematic reviews and Meta-Analyses) guidelines checklist.

Multimedia Appendix 3 Basic characteristics of the included studies.

Multimedia Appendix 4 The TIDieR (Template for Intervention Description and Replication) checklist for the included studies.

Multimedia Appendix 5 Methodological quality of each randomized controlled trial and quasi-experimental study.

Multimedia Appendix 6 Main outcome indicators and intervention effects of the included studies.

## Abbreviations

6-MWD: 6-minute walk distance
CONSORT: Consolidated Standards of Reporting Trails
FEV1: forced expiratory volume in 1 second
FVC: forced vital capacity
LC: lung cancer
mMRC: modified Medical Research Council
PPC: postoperative pulmonary complication
PR: pulmonary rehabilitation
PRISMA: Preferred Reporting Items for Systematic reviews and Meta-Analyses
RCT: randomized controlled trial
TIDieR: Template for Intervention Description and Replication
TREND: Transparent Reporting of Evaluations with Nonrandomized Designs
