# Supplementary material for: Effectiveness and Feasibility of Digital Pulmonary Rehabilitation in Patients Undergoing Lung Cancer Surgery: Systematic Review and Meta-Analysis
Source: J Med Internet Res. 2024 Nov 11;26:e56795. doi: 10.2196/56795 (PMC11589499; doi:10.2196/56795)
Supplement: Multimedia Appendix 1 [file jmir_v26i1e56795_app1.docx]

**Multimedia Appendix 1**

**TableS1 Search strategies in each included database**

| CNKI | | |
| --- | --- | --- |
| 1/1/2024 | | |
| #1 | （主题：肺癌 + 肺肿瘤 + 肺腺癌 + 非小细胞肺癌 + 肺恶性肿瘤 + 支气管肺癌 + 原发性支气管癌 + 鳞癌（精确）） | 229,202 |
| #2 | (主题:信息化 + 大数据 + Web + 机器人 + 视频 + 线上 + 互联网 + App + 微课 + 微信 + 网络 + 移动 + 远程 + 数字化 + 软件 + 平台 + 公众号 + 混合式教育 + 钉钉 + QQ + 腾讯会议(精确)) OR (主题:小程序 + 推特 + 抖音 + 新媒体 + 学习通 + 智慧树 + 学堂在线 + 应用程序(精确)) | 356,059 |
| #3 | （主题：肺康复 + 呼吸锻炼 + 呼吸康复 + 肺功能锻炼 + 运动训练 + 呼吸训练 + 肺功能锻炼 + 肺功能训练 + 加速康复外科 + 快速康复 + ERAS（精确）） | 181,991 |
| #4 | （主题：围手术期 + 术前 + 术后 + 围术期 + 肺切除术 + 肺癌根治术 + 肺叶切除术 + 肺楔形切除术 + 全肺切除术 + 肺袖式切除术 + 肺段切除术 + 胸腔镜手术 + 开胸手术（精确）） | 1,353,489 |
| #5 | #1 AND #2 AND #3 AND #4 | 514 |
| VIP | | |
| 1/1/2024 | | |
| #1 | 题名或关键词=肺癌 + 肺肿瘤 + 肺腺癌 + 非小细胞肺癌 + 肺恶性肿瘤 + 支气管肺癌 + 原发性支气管癌 + 鳞癌 | 152,767 |
| #2 | 题名或关键词=信息化 + 大数据 + Web + 机器人 + 视频 + 线上 + 互联网 + App + 微课 + 微信 + 网络 + 移动 + 远程 + 数字化 + 软件 + 平台 + 公众号 + 混合式教育 + 钉钉 + QQ + 腾讯会议 OR 题名或关键词=小程序 + 推特 + 抖音 + 新媒体 + 学习通 + 智慧树 + 学堂在线 + 应用程序 | 3,875,561 |
| #3 | 题名或关键词=肺康复 + 呼吸锻炼 + 呼吸康复 + 运动训练 + 呼吸训练 + 肺功能锻炼 + 肺功能训练 + 加速康复外科 + 快速康复 + ERAS | 47,702 |
| #4 | 题名或关键词=围手术期 + 术前 + 术后 + 围术期 + 肺切除术 + 肺癌根治术 + 肺叶切除术 + 肺楔形切除术 + 全肺切除术 + 肺袖式切除术 + 肺段切除术 + 胸腔镜手术 + 开胸手术 | 505,519 |
| #5 | #1 AND #2 AND #3 AND #4 | 27 |
| WanFang Data | | |
| 1/1/2024 | | |
| #1 | 主题:(肺癌 OR 肺肿瘤 OR 肺腺癌 OR 非小细胞肺癌 OR 肺恶性肿瘤 OR 支气管肺癌 OR 原发性支气管癌 OR 鳞癌) | 317,137 |
| #2 | 主题:(信息化 OR 大数据 OR Web OR 机器人 OR 视频 OR 线上 OR 互联网 OR App OR 微课 OR 微信 OR 网络 OR 移动 OR 远程 OR 数字化 OR 软件 OR 平台) or 主题:(公众号 OR 混合式教育 OR 钉钉 OR QQ OR 腾讯会议 OR 小程序 OR 推特 OR 抖音 OR 新媒体 OR 学习通 OR 智慧树 OR 学堂在线 OR 应用程序) | 14,520,843 |
| #3 | 主题:(肺康复 OR 呼吸锻炼 OR 呼吸康复 OR 肺功能锻炼 OR 运动训练 OR 呼吸训练 OR 肺功能锻炼 OR 肺功能训练 OR 加速康复外科 OR 快速康复 OR ERAS) | 143,802 |
| #4 | 主题:(围手术期 OR 术前 OR 术后 OR 围术期 OR 肺切除术 OR 肺癌根治术 OR 肺叶切除 OR 肺楔形切除术 OR 全肺切除术 OR 肺袖式切除术 OR 肺段切除术 OR 胸腔镜手术 OR 开胸手术) | 20,597,6020 |
| #5 | 主题:(肺癌 OR 肺肿瘤 OR 肺腺癌 OR 非小细胞肺癌 OR 肺恶性肿瘤 OR 支气管肺癌 OR 原发性支气管癌 OR 鳞癌) and 主题:(肺康复 OR 呼吸锻炼 OR 呼吸康复 OR 肺功能锻炼 OR 运动训练 OR 呼吸训练 OR 肺功能锻炼 OR 肺功能训练 OR 加速康复外科 OR 快速康复 OR ERAS) and 主题:(围手术期 OR 术前 OR 术后 OR 围术期 OR 肺切除术 OR 肺癌根治术 OR 肺叶切除 OR 肺楔形切除术 OR 全肺切除术 OR 肺袖式切除术 OR 肺段切除术 OR 胸腔镜手术 OR 开胸手术) and 主题:(信息化 OR 大数据 OR Web OR 机器人 OR 视频 OR 线上 OR 互联网 OR App OR 微课 OR 微信 OR 网络 OR 移动 OR 远程 OR 数字化 OR 软件 OR 平台 OR 公众号 OR 混合式教育 OR 钉钉 OR QQ OR 腾讯会议 OR 小程序 OR 推特 OR 抖音 OR 新媒体 OR 学习通 OR 智慧树 OR 学堂在线 OR 应用程序) | 372 |
| CBM | | |
| 1/1/2024 | | |
| #1 | ( “肺癌”[常用字段:智能] OR “肺肿瘤”[常用字段:智能] OR “肺腺癌”[常用字段:智能] OR “非小细胞肺癌”[常用字段:智能] OR “肺恶性肿瘤”[常用字段:智能] OR “支气管肺癌”[常用字段:智能] OR “原发性支气管癌”[常用字段:智能] OR “鳞癌”[常用字段:智能]) | [192602](javascript:void(0);) |
| #2 | ( “信息化”[常用字段:智能] OR “大数据”[常用字段:智能] OR “Web”[常用字段:智能] OR “机器人”[常用字段:智能] OR “视频”[常用字段:智能] OR “线上”[常用字段:智能] OR “互联网”[常用字段:智能] OR “App”[常用字段:智能] OR “微课”[常用字段:智能] OR “微信”[常用字段:智能] OR “网络”[常用字段:智能] OR “移动”[常用字段:智能] OR “远程”[常用字段:智能] OR “数字化”[常用字段:智能] OR “软件”[常用字段:智能] OR “平台”[常用字段:智能]) OR( “公众号”[常用字段:智能] OR “混合式教育”[常用字段:智能] OR “钉钉”[常用字段:智能] OR “QQ”[常用字段:智能] OR “腾讯会议”[常用字段:智能] OR “小程序”[常用字段:智能] OR “推特”[常用字段:智能] OR “抖音”[常用字段:智能] OR “新媒体”[常用字段:智能] OR “学习通”[常用字段:智能] OR “智慧树”[常用字段:智能] OR “学堂在线”[常用字段:智能] OR “应用程序”[常用字段:智能]) | [564440](javascript:void(0);) |
| #3 | ( “肺康复”[常用字段:智能] OR “呼吸锻炼”[常用字段:智能] OR “呼吸康复”[常用字段:智能] OR “肺功能锻炼”[常用字段:智能] OR “运动训练”[常用字段:智能] OR “呼吸训练”[常用字段:智能] OR “肺功能锻炼”[常用字段:智能] OR “肺功能训练”[常用字段:智能] OR “加速康复外科”[常用字段:智能] OR “快速康复”[常用字段:智能] OR “ERAS”[常用字段:智能]) | [132204](javascript:void(0);) |
| #4 | ( “围手术期”[常用字段:智能] OR “术前”[常用字段:智能] OR “术后”[常用字段:智能] OR “围术期”[常用字段:智能] OR “肺切除术”[常用字段:智能] OR “肺癌根治术”[常用字段:智能] OR “肺叶切除”[常用字段:智能] OR “肺楔形切除术”[常用字段:智能] OR “全肺切除术”[常用字段:智能] OR “肺袖式切除术”[常用字段:智能] OR “肺段切除术”[常用字段:智能] OR “胸腔镜手术”[常用字段:智能] OR “开胸手术”[常用字段:智能]) | [1586448](javascript:void(0);) |
| #5 | #1 AND #2 AND #3 AND #4 | 90 |
| PubMed | | |
| 1/1/2024 | | |
| #1 | “Lung Neoplasms”[Text Word] OR “Carcinoma, Non-Small-Cell Lung”[Text Word] OR “pulmonary neoplasms”[Text Word] OR “lung neoplasm”[Text Word] OR “pulmonary neoplasm”[Text Word] OR “lung cancer”[Text Word] OR “lung cancers”[Text Word] OR “pulmonary cancer”[Text Word] OR “pulmonary cancers”[Text Word] OR “cancer of the lung”[Text Word] OR “cancer of lung”[Text Word] OR “lung carcinoma”[Text Word] OR “lung tumor”[Text Word] OR “adenocarcinoma of lung”[Text Word] OR “bronchial neoplasms”[Text Word] OR “pulmonary tumor*”[Text Word] OR “pulmonary carcinoma*”[Text Word] | 353,693 |
| #2 | “Information Technology”[Text Word] OR “Big Data”[Text Word] OR “Internet”[Text Word] OR “Social Media”[Text Word] OR Online[Text Word] OR “Web Browser”[Text Word] OR Software[Text Word] OR “Mobile Applications”[Text Word] OR Smartphone[Text Word] OR “Cell Phone”[Text Word] OR informatization[Text Word] OR WeChat[Text Word] OR “cloud platform”[Text Word] OR informationization[Text Word] OR “information-based”[Text Word] OR App[Text Word] OR video[Text Word] OR “micro-lecture”[Text Word] OR mobile[Text Word] OR telemedicine[Text Word] OR network[Text Word] OR visualization[Text Word] OR digital[Text Word] OR remote[Text Word] OR “distance education”[Text Word] OR Telerehabilitation[Text Word] OR “virtual visit”[Text Word] OR “virtual appointment”[Text Word] OR “video visit”[Text Word] OR “online visit”[Text Word] OR “online appointment”[Text Word] OR “e-consultation”[Text Word] OR “remote consultation”[Text Word] OR “remote consult”[Text Word] OR teleconsultation[Text Word] OR “teleconsult”[Text Word] OR “videoconference”[Text Word] OR “wearables”[Text Word] OR “mobile sensing”[Text Word] OR “mobile health”[Text Word] OR “smartwatch*”[Text Word] OR “eHealth”[Text Word] OR “mHealth”[Text Word] OR telephone*[Text Word] OR technolog*[Text Word] OR telenursing[Text Word] OR “technology-based”[Text Word] OR “Chat GPT”[Text Word] OR “text message*”[Text Word] OR QQ[Text Word] OR “official account”[Text Word] OR “Blended education”[Text Word] OR “Ding Talk”[Text Word] OR tweet[Text Word] OR Microblog[Text Word] OR “Tik Tok”[Text Word] OR Kuai Shou[Text Word] | 2,550,046 |
| #3 | “Pulmonary rehabilitation”[Text Word] OR “Lung rehabilitation”[Text Word] OR “exercise training”[Text Word] OR “exercise therapy”[Text Word] OR “physical therapy”[Text Word] OR Rehabilitation[Text Word] OR “Respiratory Therapy”[Text Word] OR physiotherapy[Text Word] OR “Pulmonary exercise”[Text Word] OR “breathing exercise”[Text Word] OR “respiratory rehabilitation”[Text Word] OR “fast-track surgery”[Text Word] OR “accelerated rehabilitation”[Text Word] OR “ERAS”[Text Word] OR “Exercise endurance”[Text Word] OR “Postoperative rehabilitation”[Text Word] OR “multimodal rehabilitation”[Text Word] OR “FTS”[Text Word] OR “enhanced recovery”[Text Word] OR “ERAS program”[Text Word] | 486,770 |
| #4 | “Perioperative Period*”[Text Word] OR “Period, Perioperative”[Text Word] OR “Periods, Perioperative”[Text Word] OR pneumonectomy[Text Word] OR lobectomy[Text Word] OR postoperative[Text Word] OR preoperative OR “pulmonary lobectomy”[Text Word] OR “thoracic surgery”[Text Word] OR “multimodal perioperative care”[Text Word] OR bilobectomy[Text Word] OR “pulmonary resection”[Text Word] OR “Radical operation”[Text Word] OR “pulmonary wedge resection”[Text Word] OR “sleeve lobectomy”[Text Word] OR VATS[Text Word] | 1,287,698 |
| #5 | #1 AND #2 AND #3 AND #4 | 179 |
| Web of science | | |
| 1/1/2024 | | |
| #1 | ((((((((((((((((TS=(“Lung Neoplasms”)) OR TS=(“Carcinoma, Non-Small-Cell Lung”)) OR TS=(“pulmonary neoplasms”)) OR TS=(“lung neoplasm”)) OR TS=(“pulmonary neoplasm”)) OR TS=(“lung cancer”)) OR TS=(“lung cancers”)) OR TS=(“pulmonary cancer”)) OR TS=(“pulmonary cancers”)) OR TS=(“cancer of the lung”)) OR TS=(“cancer of lung”)) OR TS=(“lung carcinoma”)) OR TS=(“lung tumor”)) OR TS=(“adenocarcinoma of lung”)) OR TS=(“bronchial neoplasms”)) OR TS=(“pulmonary tumor*”)) OR TS=(“pulmonary carcinoma*”) | 352,130 |
| #2 | TS=(“Information Technology” OR “Big Data” OR “Internet” OR “Social Media” OR Online OR “Web Browser” OR Software OR “Mobile Applications” OR Smartphone OR “Cell Phone” OR informatization OR WeChat OR “cloud platform” OR informationization OR “information-based” OR App OR video OR “micro-lecture” OR mobile OR telemedicine OR network OR visualization OR digital OR remote OR “distance education” OR Telerehabilitation OR “virtual visit” OR “virtual appointment” OR “video visit” OR “online visit” OR “online appointment” OR “e-consultation” OR “remote consultation” OR “remote consult” OR teleconsultation OR “teleconsult” OR “videoconference” OR “wearables” OR “mobile sensing” OR “mobile health” OR “smartwatch*” OR “eHealth” OR “mHealth” OR telephone* OR technolog* OR telenursing OR “technology-based” OR “Chat GPT” OR “text message*” OR QQ OR “official account” OR “Blended education” OR “Ding Talk” OR tweet OR Microblog OR “Tik Tok” OR Kuai Shou) | 8,579,078 |
| #3 | [TS=(“Pulmonary rehabilitation” OR “Lung rehabilitation” OR “exercise training” OR “exercise therapy” OR “physical therapy” OR Rehabilitation OR “Respiratory Therapy” OR physiotherapy OR “Pulmonary exercise” OR “breathing exercise” OR “respiratory rehabilitation” OR “fast-track surgery” OR “accelerated rehabilitation” OR “ERAS” OR “Exercise endurance” OR “Postoperative rehabilitation” OR “multimodal rehabilitation” OR “FTS” OR “enhanced recovery” OR “ERAS program”)](https://www.webofscience.com/wos/alldb/summary/ee6f7f5f-8266-424f-a797-90c808392f0b-9272df54/relevance/1) | 370,501 |
| #4 | TS=(“Perioperative Period*” OR “Period, Perioperative” OR “Periods, Perioperative” OR pneumonectomy OR lobectomy OR postoperative OR preoperative OR “pulmonary lobectomy” OR “thoracic surgery” OR “multimodal perioperative care” OR bulbectomy OR “pulmonary resection” OR “Radical operation” OR “pulmonary wedge resection” OR “sleeve lobectomy” OR VATS) | 722,639 |
| #5 | #1 AND #2 AND #3 AND #4 | 195 |
| Cochrane library | | |
| 1/1/2024 | | |
| #1 | (“Lung Neoplasms” OR “Carcinoma, Non-Small-Cell Lung” OR “pulmonary neoplasms” OR “lung neoplasm” OR “pulmonary neoplasm” OR “lung cancer” OR “lung cancers” OR “pulmonary cancer” OR “pulmonary cancers” OR “cancer of the lung” OR “cancer of lung” OR “lung carcinoma” OR “lung tumor” OR “adenocarcinoma of lung” OR “bronchial neoplasms” OR “pulmonary tumor*” OR “pulmonary carcinoma*”): ti, ab, kw | 27043 |
| #2 | (“Information Technology” OR “Big Data” OR “Internet” OR “Social Media” OR Online OR “Web Browser” OR Software OR “Mobile Applications” OR Smartphone OR “Cell Phone” OR informatization OR WeChat OR “cloud platform” OR informationization OR “information-based” OR App OR video OR “micro-lecture” OR mobile OR telemedicine OR network OR visualization OR digital OR remote OR “distance education” OR Telerehabilitation OR “virtual visit” OR “virtual appointment” OR “video visit” OR “online visit” OR “online appointment” OR “e-consultation” OR “remote consultation” OR “remote consult” OR teleconsultation OR “teleconsult” OR “videoconference” OR “wearables” OR “mobile sensing” OR “mobile health” OR “smartwatch*” OR “eHealth” OR “mHealth” OR telephone* OR technolog* OR telenursing OR “technology-based” OR “Chat GPT” OR “text message*” OR QQ OR “official account” OR “Blended education” OR “Ding Talk” OR tweet OR Microblog OR “Tik Tok” OR Kuai Shou): ti, ab, kw | 334219 |
| #3 | (“Pulmonary rehabilitation” OR “Lung rehabilitation” OR “exercise training” OR “exercise therapy” OR “physical therapy” OR Rehabilitation OR “Respiratory Therapy” OR physiotherapy OR “Pulmonary exercise” OR “breathing exercise” OR “respiratory rehabilitation” OR “fast-track surgery” OR “accelerated rehabilitation” OR “ERAS” OR “Exercise endurance” OR “Postoperative rehabilitation” OR “multimodal rehabilitation” OR “FTS” OR “enhanced recovery” OR “ERAS program”): ti, ab, kw | 107885 |
| #4 | (“Perioperative Period*” OR “Period, Perioperative” OR “Periods, Perioperative” OR pneumonectomy OR lobectomy OR postoperative OR preoperative OR “pulmonary lobectomy” OR “thoracic surgery” OR “multimodal perioperative care” OR bilobectomy OR “pulmonary resection” OR “Radical operation” OR “pulmonary wedge resection” OR “sleeve lobectomy” OR VATS): ti, ab, kw | 181660 |
| #5 | #1 AND #2 AND #3 AND #4 | 128 |
| Embase | | |
| 1/1/2024 | | |
| #1 | 'lung neoplasms': ti, ab, kw OR 'carcinoma, non-small-cell lung': ti, ab, kw OR 'pulmonary neoplasms': ti, ab, kw OR 'lung neoplasm': ti, ab, kw OR 'pulmonary neoplasm': ti, ab, kw OR 'lung cancer': ti, ab, kw OR 'lung cancers': ti, ab, kw OR 'pulmonary cancer': ti, ab, kw OR 'pulmonary cancers': ti, ab, kw OR 'cancer of the lung': ti, ab, kw OR 'cancer of lung': ti, ab, kw OR 'lung carcinoma': ti, ab, kw OR 'lung tumor': ti, ab, kw OR 'adenocarcinoma of lung': ti, ab, kw OR 'bronchial neoplasms': ti, ab, kw OR 'pulmonary tumor*': ti, ab, kw OR 'pulmonary carcinoma*': ti, ab, kw | 356683 |
| #2 | 'information technology': ti, ab, kw OR 'big data': ti, ab, kw OR 'internet': ti, ab, kw OR 'social media': ti, ab, kw OR online: ti, ab, kw OR 'web browser': ti, ab, kw OR software: ti, ab, kw OR 'mobile applications': ti, ab, kw OR smartphone: ti, ab, kw OR 'cell phone': ti, ab, kw OR informatization: ti, ab, kw OR WeChat: ti, ab, kw OR 'cloud platform': ti, ab, kw OR informationization: ti, ab, kw OR 'information-based': ti, ab, kw OR app: ti, ab, kw OR video: ti, ab, kw OR 'micro-lecture': ti, ab, kw OR mobile: ti, ab, kw OR telemedicine: ti, ab, kw OR network: ti, ab, kw OR visualization: ti, ab, kw OR digital: ti, ab, kw OR remote: ti, ab, kw OR 'distance education': ti, ab, kw OR telerehabilitation: ti, ab, kw OR 'virtual visit': ti, ab, kw OR 'virtual appointment': ti, ab, kw OR 'video visit': ti, ab, kw OR 'online visit': ti, ab, kw OR 'online appointment': ti, ab, kw OR 'e-consultation': ti, ab, kw OR 'remote consultation': ti, ab, kw OR 'remote consult': ti, ab, kw OR teleconsultation: ti, ab, kw OR 'teleconsult': ti, ab, kw OR 'videoconference': ti, ab, kw OR 'wearables': ti, ab, kw OR 'mobile sensing': ti, ab, kw OR 'mobile health': ti, ab, kw OR 'smartwatch*': ti, ab, kw OR 'ehealth': ti, ab, kw OR 'mhealth': ti, ab, kw OR telephone*: ti, ab, kw OR technolog*: ti, ab, kw OR telenursing: ti, ab, kw OR 'technology-based': ti, ab, kw OR 'chat gpt': ti, ab, kw OR 'text messag*': ti, ab, kw OR qq: ti, ab, kw OR 'official account': ti, ab, kw OR 'blended education': ti, ab, kw OR 'ding talk': ti, ab, kw OR tweet: ti, ab, kw OR microblog: ti, ab, kw OR 'tik tok': ti, ab, kw OR Kuai Shou: ti, ab, kw | 3086726 |
| #3 | ('pulmonary rehabilitation': ti, ab, kw OR 'lung rehabilitation': ti, ab, kw OR 'exercise training': ti, ab, kw OR 'exercise therapy': ti, ab, kw OR 'physical therapy modalities': ti, ab, kw OR rehabilitation: ti, ab, kw OR 'respiratory therapy': ti, ab, kw OR physiotherapy: ti, ab, kw OR 'pulmonary exercise': ti, ab, kw OR 'breathing exercise': ti, ab, kw OR 'respiratory rehabilitation': ti, ab, kw OR 'fast track surgery': ti, ab, kw OR 'accelerated rehabilitation': ti, ab, kw OR 'eras': ti, ab, kw OR 'exercise endurance': ti, ab, kw OR 'postoperative rehabilitation': ti, ab, kw OR 'multimodal rehabilitation': ti, ab, kw OR 'fast track': ti, ab, kw OR 'fts': ti, ab, kw OR 'enhanced recovery': ti, ab, kw OR 'ERAS program': ti, ab, kw) | 429336 |
| #4 | 'perioperative period*': ti, ab, kw OR 'period, perioperative': ti, ab, kw OR 'periods, perioperative': ti, ab, kw OR pneumonectomy: ti, ab, kw OR lobectomy: ti, ab, kw OR postoperative: ti, ab, kw OR 'preoperative or pulmonary lobectomy': ti, ab, kw OR 'thoracic surgery': ti, ab, kw OR 'multimodal perioperative care': ti, ab, kw OR bilobectomy: ti, ab, kw OR 'pulmonary resection': ti, ab, kw OR 'radical operation': ti, ab, kw OR 'pulmonary wedge resection': ti, ab, kw OR 'sleeve lobectomy': ti, ab, kw OR VATS: ti, ab, kw | 1258354 |
| #5 | #1 AND #2 AND #3 AND #4 | 194 |
| Medline | | |
| 1/1/2024 | | |
| #1 | TX (“Lung Neoplasms” OR “Carcinoma, Non-Small-Cell Lung” OR “pulmonary neoplasms” OR “lung neoplasm” OR “pulmonary neoplasm” OR “lung cancer” OR “lung cancers” OR “pulmonary cancer” OR “pulmonary cancers” OR “cancer of the lung” OR “cancer of lung” OR “lung carcinoma” OR “lung tumor” OR “adenocarcinoma of lung” OR “bronchial neoplasms” OR “pulmonary tumor*” OR “pulmonary carcinoma*”) | 234,283 |
| #2 | TX “Information Technology” OR “Big Data” OR “Internet” OR “Social Media” OR Online OR “Web Browser” OR Software OR “Mobile Applications” OR Smartphone OR “Cell Phone” OR informatization OR WeChat OR “cloud platform” OR informationization OR “information-based” OR App OR video OR “micro-lecture” OR mobile OR telemedicine OR network OR visualization OR digital OR remote OR “distance education” OR Telerehabilitation OR “virtual visit” OR “virtual appointment” OR “video visit” OR “online visit” OR “online appointment” OR “e-consultation” OR “remote consultation” OR “remote consult” OR teleconsultation OR “teleconsult” OR “videoconference” OR “wearables” OR “mobile sensing” OR “mobile health” OR “smartwatch*” OR “eHealth” OR “mHealth” OR telephone* OR technolog* OR telenursing OR “technology-based” OR “Chat GPT” OR “text message*” OR QQ OR “official account” OR “Blended education” OR “Ding Talk” OR tweet OR Microblog OR “Tik Tok” OR Kuai Shou | 3,224,352 |
| #3 | TX ( “Pulmonary rehabilitation” OR “Lung rehabilitation” OR “exercise training” OR “exercise therapy” OR “physical therapy” OR Rehabilitation OR “Respiratory Therapy” OR physiotherapy OR “Pulmonary exercise” OR “breathing exercise” OR “respiratory rehabilitation” OR “fast-track surgery” OR “accelerated rehabilitation” OR “ERAS” OR “Exercise endurance” OR “Postoperative rehabilitation” OR “multimodal rehabilitation” OR “FTS” OR “enhanced recovery” OR “ERAS program” ) | 433,142 |
| #4 | TX (“Perioperative Period*” OR “Period, Perioperative” OR “Periods, Perioperative” OR pneumonectomy OR lobectomy OR postoperative OR preoperative OR “pulmonary lobectomy” OR “thoracic surgery” OR “multimodal perioperative care” OR bilobectomy OR “pulmonary resection” OR “Radical operation” OR “pulmonary wedge resection” OR “sleeve lobectomy” OR VATS) | 722,463 |
| #5 | #1 AND #2 AND #3 AND #4 | 461 |

| CINAHL | | |
| --- | --- | --- |
| 1/1/2024 | | |
| #1 | SU (“Lung Neoplasms” OR “Carcinoma, Non-Small-Cell Lung” OR “pulmonary neoplasms” OR “lung neoplasm” OR “pulmonary neoplasm” OR “lung cancer” OR “lung cancers” OR “pulmonary cancer” OR “pulmonary cancers” OR “cancer of the lung” OR “cancer of lung” OR “lung carcinoma” OR “lung tumor” OR “adenocarcinoma of lung” OR “bronchial neoplasms” OR “pulmonary tumor*” OR “pulmonary carcinoma*”) | 36,371 |
| #2 | SU “Information Technology” OR “Big Data” OR “Internet” OR “Social Media” OR Online OR “Web Browser” OR Software OR “Mobile Applications” OR Smartphone OR “Cell Phone” OR informatization OR WeChat OR “cloud platform” OR informationization OR “information-based” OR App OR video OR “micro-lecture” OR mobile OR telemedicine OR network OR visualization OR digital OR remote OR “distance education” OR Telerehabilitation OR “virtual visit” OR “virtual appointment” OR “video visit” OR “online visit” OR “online appointment” OR “e-consultation” OR “remote consultation” OR “remote consult” OR teleconsultation OR “teleconsult” OR “videoconference” OR “wearables” OR “mobile sensing” OR “mobile health” OR “smartwatch*” OR “eHealth” OR “mHealth” OR telephone* OR technolog* OR telenursing OR “technology-based” OR “Chat GPT” OR “text message*” OR QQ OR “official account” OR “Blended education” OR “Ding Talk” OR tweet OR Microblog OR “Tik Tok” OR Kuai Shou | 487,555 |
| #3 | SU (“Pulmonary rehabilitation” OR “Lung rehabilitation” OR “exercise training” OR “exercise therapy” OR “physical therapy” OR Rehabilitation OR “Respiratory Therapy” OR physiotherapy OR “Pulmonary exercise” OR “breathing exercise” OR “respiratory rehabilitation” OR “fast-track surgery” OR “accelerated rehabilitation” OR “ERAS” OR “Exercise endurance” OR “Postoperative rehabilitation” OR “multimodal rehabilitation” OR “FTS” OR “enhanced recovery” OR “ERAS program”) | 115,314 |
| #4 | SU (“Perioperative Period*” OR “Period, Perioperative” OR “Periods, Perioperative” OR pneumonectomy OR lobectomy OR postoperative OR preoperative OR “pulmonary lobectomy” OR “thoracic surgery” OR “multimodal perioperative care” OR bilobectomy OR “pulmonary resection” OR “Radical operation” OR “pulmonary wedge resection” OR “sleeve lobectomy” OR VATS) | 109,921 |
| #5 | #1 AND #2 AND #3 AND #4 | 20 |

| **Total：2180** |
| --- |

**TableS2 Search terms list**

| **Category** | **Search terms** |
| --- | --- |
| Lung Neoplasms | “Lung Neoplasms” OR “Carcinoma, Non-Small-Cell Lung” OR “pulmonary neoplasms” OR “lung neoplasm” OR “pulmonary neoplasm” OR “lung cancer” OR “lung cancers” OR “pulmonary cancer” OR “pulmonary cancers” OR “cancer of the lung” OR “cancer of lung” OR “lung carcinoma” OR “lung tumor” OR “adenocarcinoma of lung” OR “bronchial neoplasms” OR “pulmonary tumor*” OR “pulmonary carcinoma*” |
| Digital | “Information Technology” OR “Big Data” OR “Internet” OR “Social Media” OR Online OR “Web Browser” OR Software OR “Mobile Applications” OR Smartphone OR “Cell Phone” OR informatization OR WeChat OR “cloud platform” OR informationization OR “information-based” OR App OR video OR “micro-lecture” OR mobile OR telemedicine OR network OR visualization OR digital OR remote OR “distance education” OR Telerehabilitation OR “virtual visit” OR “virtual appointment” OR “video visit” OR “online visit” OR “online appointment” OR “e-consultation” OR “remote consultation” OR “remote consult” OR teleconsultation OR “teleconsult” OR “videoconference” OR “wearables” OR “mobile sensing” OR “mobile health” OR “smartwatch*” OR “eHealth” OR “mHealth” OR telephone* OR technolog* OR telenursing OR “technology-based” OR “Chat GPT” OR “text message*” OR QQ OR “official account” OR “Blended education” OR “Ding Talk” OR tweet OR Microblog OR “Tik Tok” OR Kuai Shou |
| Pulmonary rehabilitation | “Pulmonary rehabilitation” OR “Lung rehabilitation” OR “exercise training” OR “exercise therapy” OR “physical therapy” OR Rehabilitation OR “Respiratory Therapy” OR physiotherapy OR “Pulmonary exercise” OR “breathing exercise” OR “respiratory rehabilitation” OR “fast-track surgery” OR “accelerated rehabilitation” OR “ERAS” OR “Exercise endurance” OR “Postoperative rehabilitation” OR “multimodal rehabilitation” OR “FTS” OR “enhanced recovery” OR “ERAS program” |
| Preoperative and postoperative | “Perioperative Period*” OR “Period, Perioperative” OR “Periods, Perioperative” OR pneumonectomy OR lobectomy OR postoperative OR preoperative OR “pulmonary lobectomy” OR “thoracic surgery” OR “multimodal perioperative care” OR bilobectomy OR “pulmonary resection” OR “Radical operation” OR “pulmonary wedge resection” OR “sleeve lobectomy” OR VATS |

**TableS3 Search terms list**

| **Category** | **Search terms** |
| --- | --- |
| 肺癌 | 肺癌 + 肺肿瘤 + 肺腺癌 + 非小细胞肺癌 + 肺恶性肿瘤 + 支气管肺癌 + 原发性支气管癌 + 鳞癌 |
| 数字化 | 信息化 + 大数据 + Web + 机器人 + 视频 + 线上 + 互联网 + App + 微课 + 微信 + 网络 + 移动 + 远程 + 数字化 + 软件 + 平台 + 公众号 + 混合式教育 + 钉钉 + QQ + 腾讯会议 + 小程序 + 推特 + 抖音 + 新媒体 + 学习通 + 智慧树 + 学堂在线 + 应用程序 |
| 肺康复 | 肺康复 + 呼吸锻炼 + 呼吸康复 + 肺功能锻炼 + 运动训练 + 呼吸训练 + 肺功能锻炼 + 肺功能训练 + 加速康复外科 + 快速康复 + ERAS |
| 术前、术后 | 围手术期 + 术前 + 术后 + 围术期 + 肺切除术 + 肺癌根治术 + 肺叶切除 +肺楔形切除术 + 全肺切除术 + 肺袖式切除术 + 肺段切除术 + 胸腔镜手术 + 开胸手术 |
