# Supplementary material for: Effectiveness and Feasibility of Digital Pulmonary Rehabilitation in Patients Undergoing Lung Cancer Surgery: Systematic Review and Meta-Analysis
Source: J Med Internet Res. 2024 Nov 11;26:e56795. doi: 10.2196/56795 (PMC11589499; doi:10.2196/56795)
Supplement: Multimedia Appendix 3 [file jmir_v26i1e56795_app3.docx]

| Study; country | Study design | Setting and study group demographics | Disease characteristics, n | Eligibility criteria | |
| --- | --- | --- | --- | --- | --- |
|  |  |  |  | Inclusion criteria | Exclusion criteria |
| Ji et al [32];  Korea | RCT | •Hospitals  •IG: n=32 (24 males/8 females); mean age 60.50 (SD 10.20)  •CG: n=32 (21 males/11 females); mean age 57.97 (SD 9.87) | •LC stages (IG/CG): stage I (7/13), stage II (5/3), stage III (7/7), and stage Ⅳ (13/8)  •Risk factors (IG/CG): diabetes (5/7), hypertension (11/9), tuberculosis (7/4), previous surgery (21/28), ex-smokers (15/11), current smoker (7/8), and others (14/20)  •Surgical types: not available | •Age 20–80 years  •NSCLC  •Obstructive ventilatory disturbance  •6-MWD> 150 m  •Possession of an Android mobile phone  •Written consent | •Severe cerebrovascular or musculoskeletal disease and unable to follow the exercise  •Illiterate or limited communication ability  •Major disability may cause death≤ 1 year  •Refuse to attend |
| Sui et al [31]; China | RCT | •Hospitals  •IG: n=100 (80 males/20 females); mean age 61.37 (SD 11.21)  •CG: n=100 (84 males/16 females); mean age 62.35 (SD 9.98) | •LC stages (IG/CG): stage I (36/37), stage II (26/34), and stage III (38/29)  •Risk factors (IG/CG): hypertension (31/40), hyperlipidemia (20/27), diabetes (7/12), smoking history (57/62), drink history (36/41)  •Surgical types: NA | •Age> 18 years  •NSCLC  •Underwent surgical resection  •No difficulty in fulfilling questionnaires  •Skillful in using mobile phone  •Possible followed up | •Severe cognitive impairments  •Severe psychiatric disorders  •Other malignancies  •Uncontrolled medical disease  •Pregnant or breastfeeding women |
| Chu et al [27]; China | RCT | - •Hospitals - •IG: n=104 (43 males/61 females); mean age 55.1 (SD 9.8) - •CG: n=104 (46 males/58 females); mean age 55.5 (SD 10.1) | •LC stages (IG/CG): stage I (91/92), stage II (10/8), stage III (3/4)  •Risk factors (IG/CG): nonsmoker (72/63), smoking<15 years (15/19), smoking≥15 years (17/22)  •Surgical type (IG/CG): wedge resection (23/30), pulmonary segmentectomy (40/43), lobectomy (41/31) | •Age< 75 years  •Thoracoscopic surgery  •Pathology with stage I, II, or IIIA LC  •Kamofsky score≥ 60 | •Join another clinical trial in 4 weeks prior  •With COPD, peripheral vascular disease, neuromuscular disease  •Cardiac function class III or IV  •Lack of primary education or with cognitive impairment |
| Li et al [30]; China | RCT | - •A large tertiary medical center for thoracic surgery - •IG: n=40 (20 males/20 females); mean age 50.7 (SD 9.6) - •CG: n=40 (27 males/13 females); mean age 54.7 (SD 8.5) | •LC stages: NA  •Risk factors (IG/CG): comorbidities (15/14), ever or current smoker (14/22)  •Surgical types (IG/CG): open thoracotomy (16/21), video-assisted thoracoscopic surgery (24/19) | •Age> 18 years  •NSCLC  •Undergoing pulmonary surgery  •Medically stable comorbidity conditions  •Mandarin speaking | •Metastatic disease  •With sensory or cognitive disorder  •Unable to safe participation and successful communication |
| Liu and Pan [28]; China | RCT | - •Hospitals - •IG: n=55 (33 males/22 females); mean age 71.8 (SD 2.5) - •CG: n=55 (32 males/23 females); mean age 71.5 (SD 2.3) | •LC stages (IG/CG): stage I (34/33), stage II (21/22)  •Risk factors (IG/CG): smoking history (37/36)  •Surgical types: NA | •Age> 65 years  •LC patients  •Radical LC surgery  •Complete clinical data  •Predictive survival> 1 year  •Written consent | •With severe liver and kidney dysfunction, malignant tumor  •With aphasia, mental illness  •With metabolic abnormalities, coagulation disorders, and other diseases  •With cardiopulmonary impairment and respiratory distress.  •Poor co-operation |
| Sun et al [33]; United States | Quasi-experimental study (sequential enrollment pilot study) | - •Comprehensive cancer center - •Patients in the IG: n=19 (12 males/7 females); mean age 64.5 (SD 13.7) - •Caregivers in the IG: n=11 (2 males/9 females); mean age 59.5 (SD 15.6) - •Patients in the CG: n=19 (8 males/11 females); mean age 66.8 (SD 11.9) - •Caregivers in the CG: n=11 (2 males/9 females); mean age 60.5 (SD 13.3) | •LC stages: NA  •Risk factors (IG/CG): hypertension (6/11), coronary artery disease(cad) (1/3), prior cardiothoracic surgery (0/2), neoadjuvant chemotherapy (4/2), treated ≤6months before surgery (4/2), COPD (3/3), preoperative radiation (0/1), diabetes (3/5)  •Surgical type (IG/CG): wedge resection (8/2); lobectomy (9/16); bilobectomy (1/0); pneumonectomy (1/1) | •LC or a secondary malignancy of the lung  •English-speaking  •Scheduled to undergo surgery | No |
|  |  |  |  |  |  |
| Kadiri et al [15]; United Kingdom | Quasi-experimental study | - •A regional thoracic unit (hospital or community) - •IG: n=31; mean age 64 (SD 12) - •CG: n=34; mean age 64 (SD 12) | •LC stages: NA  •Risk factors (IG): smoking history (25); ischemic heart disease (2); COPD (9)  •Surgical types: NA | •Eligible for curative LC surgery | No |
| Finley et al [16]; United States | Quasi-experimental study  (single-arm, pre-post study) | - •N/A - •Enrolled: n=30 (13 males/17 females); mean age 67.5 (SD 10.6) - •Analysis: n=18 (8 males/10 females); mean age 68.2 (SD 5.4) | •LC stages (E/A): stage I (22/13); stage II (3/2); stage III (5/3)  •Risk factors: NA  •Pulmonary function (E/A): FEV1%, mean 74.2 (SD 18.2)/mean 75.4 (SD 18.6); FVC%, mean 86.1 (11.7)/mean 87.7 (11.5); DLCO, 78.7(SD 17.6)/mean 77.9 (SD 17.1)  •Surgical types: NA | •Age > 18 years  •Scheduled for LC surgery  •Access to Wi-Fi or cellular service, download the wearable fitness device app on a cellphone or computer  •Written consent | •Life expectancy <12 months, hospitalization  •Psychiatric diagnosis  •Exhibited exercise contraindications  •Unable to walk or complete 6MWD |
| Yang et al [29]; Korea | Quasi-experimental study  (single-arm, pre-post design) | - •Outpatient clinics - •IG: n=50 (28 males/22 females); mean age 58.0 (SD 11.9) | •LC stages (IG): stage I (27); stage II (6); stage III (12); stage IV (5)  •Risk factors (IG): hypertension (9); diabetes (5)  •Smoking status (IG): current (17); never (26); ex-smoker (7)  •Surgical types: NA | •Age 18–85 years  •Consented to participate  •Comorbid conditions relatively stable  •Willing to follow the study protocol | •Difficulty in giving feedback via a smartphone  •History of any serious illness other than LC  •Difficulty in performing rehabilitation exercises |
| Qin et al [26]; China | Quasi-experimental study  (comparative study before and after) | - •Hospitals - •IG: n=50 (22 males/28 females); mean age 60.38 (SD 9.53) - •CG: n=50 (25 males/25 females); mean age 58.46 (SD 10.45) | •LC stages: NA  •Risk factors: NA  •Surgical type (IG/CG): Segmentectomy (15/16), lobectomy (35/34) | •Age 18-70 years  •NSCLC  •Thoracoscopic radical LC surgery for the first time | •With severe cardiac, cerebral or renal disease  •Myocardial infarction (MI) or cerebrovascular accident (<1 year), stable angina pectoris, aneurysm, hemoptysis (<90 days), limb movement disorder, severe osteoarthritis, and other contraindications to exercise  •Radiotherapy before surgery  •Impaired consciousness or uncooperative |
| Chen et al [34]; China | Quasi-experimental study  (retrospective study) | - •Hospitals - •IG: n=51 (38 males/13 females); mean age 51.05 (SD 7.91) - •CG: n=49 (27 males/22 females); mean age 50.35 (SD 7.19) | •LC stages (IG/CG): phase Ia (2/2), phase Ib (24/7), phase IIa (7/15), phase IIb (8/14), phase IIIa (10/11)  •Risk factors (IG/CG): BMI (kg/m^2^), mean 20.7(SD 2.28)/mean 20.4 (2.76); smoker (39/37)  •Surgical types: NA | •Age> 18 years  •LC  • FEV_1_/FVC≤70%  •Had complete clinical, epidemiological, and laboratory data | •With unconsciousness and mental abnormalities  •Cancer metastasis  •With severe liver, kidney, other organ dysfunction  •With asthma, COPD, and respiratory failure before operation  •With malnutrition or obesity |

^a^M/F: number of male and female participants.

^b^NSCLC: non-small-cell lung cancer.

^c^E/A: enrolled sample and analytic sample.

^d^SD: standard deviation.

^e^CG/IG: control group and intervention group.

^f^LC：lung cancer.

^g^RCT: randomized controlled trials.

^h^6-MWD: 6-minute walk distance.

^i^NA: Not available.

^j^COPD: chronic obstructive pulmonary disease.

^k^VATS: Video-assisted thoracoscopic surgery.

^l^FVC: forced vital capacity.

^m^FEV_1_: forced expiratory volume in 1 second.
