# Supplementary material for: Effectiveness and Feasibility of Digital Pulmonary Rehabilitation in Patients Undergoing Lung Cancer Surgery: Systematic Review and Meta-Analysis
Source: J Med Internet Res. 2024 Nov 11;26:e56795. doi: 10.2196/56795 (PMC11589499; doi:10.2196/56795)
Supplement: Multimedia Appendix 5 [file jmir_v26i1e56795_app5.docx]

**Multimedia Appendix 5**

**Table 1. Methodological Quality of each randomized controlled trials**

| Study  (Fiest Author, year) | D1. Randomization  process | D2. Deviations from intended interventions | D3. Missing outcome data | D4. Measurement of the outcome | D5. Selection of the reported result | D6. Overall bias |
| --- | --- | --- | --- | --- | --- | --- |
| Ji et al, 2019 [32]. | High risk | High risk | High risk | Low risk | Low risk | High risk |
| Sui et al, 2020 [31]. | Low risk | Some concerns | Low risk | High risk | Low risk | High risk |
| Chu et al, 2020 [27]. | Some concerns | High risk | Low risk | Low risk | Some concerns | High risk |
| Li et al, 2021 [30]. | Some concerns | High risk | Low risk | Low risk | Low risk | High risk |
| Liu and Pan 2021 [28]. | Some concerns | High risk | Low risk | Low risk | Low risk | High risk |


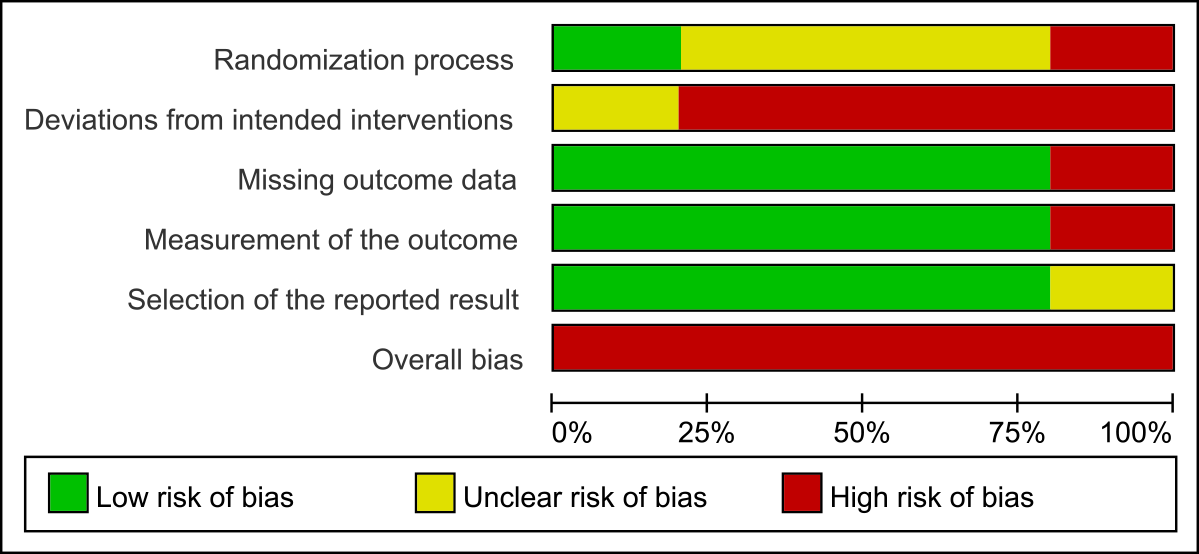


**Fig 1. Risk of bias summary-randomized controlled trials**

| Study (First Author, year) | Q1 | Q2 | Q3 | Q4 | Q5 | Q6 | Q7 | Q8 | Q9 | Total |
| --- | --- | --- | --- | --- | --- | --- | --- | --- | --- | --- |
| Qin et al, 2023 [26]. | YES | YES | YES | YES | NO | YES | Unclear | Unclear | YES | 6 |
| Sun et al, 2017 [33]. | YES | NO | Unclear | YES | YES | YES | YES | YES | YES | 7 |
| Chen et al, 2023 [34]. | YES | YES | YES | YES | YES | NO | YES | YES | YES | 8 |
| Finley et al, 2021 [16]. | YES | YES | YES | NO | YES | YES | YES | YES | YES | 8 |
| Kadiri et al, 2019 [15]. | YES | Unclear | YES | YES | NO | YES | NO | Unclear | NO | 4 |
| Yang et al, 2022 [29]. | YES | YES | YES | NO | YES | YES | YES | Unclear | NO | 6 |
| **Note:**  ^a^Q1. Is it clear in the study what is the ‘cause’ and what is the ‘effect’ (i.e. there is no confusion about which variable comes first)?  ^b^Q2.Were the participants included in any comparisons similar?  ^c^Q3.Were the participants included in any comparisons receiving similar treatment/care, other than the exposure or intervention of interest?  ^d^Q4.Was there a control group?  ^e^Q5.Were there multiple measurements of the outcome both pre and post the intervention/exposure?  ^f^Q6. Was follow up complete and if not, were differences between groups in terms of their follow up adequately described and analyzed?  ^g^Q7. Were the outcomes of participants included in any comparisons measured in the same way?  ^h^Q8. Were outcomes measured in a reliable way?  ^i^Q9. Was appropriate statistical analysis used? | | | | | | | | | | |

**Table 2. Risk of Bias in each Quasi-Experimental Studies.**
