# Supplementary material for: Effectiveness and Feasibility of Digital Pulmonary Rehabilitation in Patients Undergoing Lung Cancer Surgery: Systematic Review and Meta-Analysis
Source: J Med Internet Res. 2024 Nov 11;26:e56795. doi: 10.2196/56795 (PMC11589499; doi:10.2196/56795)
Supplement: Multimedia Appendix 6 [file jmir_v26i1e56795_app6.docx]

| Study | Outcomes(measures) | Times of evaluation | Main results | Other details (IG^a^/CG^b^) |
| --- | --- | --- | --- | --- |
| Ji et al [32] | •QOL^c^ (Euro Quality of Life-Visual Analog Scale), Euro Quality of Life 5 Dimensions Questionnaire  •Patients’ satisfaction (Patient Global Assessment)  •6-MWD^d^  •Modified Medical Research Council scores | •Baseline  •6 weeks  •12 weeks | •All participants showed improvement in 6-MWD, mMRC^e^ scores, quality of life, and patient satisfaction compared with baseline (*P<*.05). However, there were no significant differences between the 2 groups in the above indicators. | •64/158 (40.5%) screened patients were enrolled. |
| Sui et al [31] | •Anxiety (HADS-A^f^)  •Depression (HADS-D^g^)  •QOL: European Organization for Research and Treatment of Cancer Core Quality of Life Questionnaire  •Disease-free survival  •Overall survival | •At baseline (T0)  •3, 6, 9, and 12 months after initiation (T1, T2, T3, and T4)  •At 48-month follow-up (T5) | •IG had lower HADS-A and HADS-D scores and anxiety and depression rates and less 12- and 60-month follow-up loss than CG (*P<*.05).  •The general and functioning scores of the QLQ-C30 were higher than those of the CG (*P<*.05).  •The QLQ-C30 symptom scores were similar between the 2 groups and limited the survival benefit of WeChat app-based education and  rehabilitation program compared with CG. | •Patients who withdrew: 10%/20%  Intervention period (3/10)  •Nonintervention stage (7/10) |
| Chu et al [27] | •PPCs^h^ within 30 days after surgery  •Postoperative hospitalization days  •Chest drainage tube duration | •At discharge (T0)  •30 days after surgery (T1) | •IG had shorter postoperative hospital days, chest drainage duration, and a lower overall incidence of PPCs than CG (*P<*.05). However, the difference in the incidence of pulmonary atelectasis and pleural effusion was not statistically significant (*P*> .05). | •None withdrew |
| Li et al [30] | •Training knowledge  •Respiratory exercise compliance (The Pulmonary Functional Exercise Compliance Questionnaire)  •Pulmonary function: arterial partial pressure of oxygen, arterial partial pressure of carbon dioxide, breath-hold test time  •6-MWD (pedometer)  •PPCs by the Melbourne Group Scale  • Breath holding time |  | •IG exhibited lower rates of PPCs and chest drainage duration than CG (*P<*.05).  •IG demonstrated a longer 6-MWD and higher scores in knowledge and exercise adherence than CG (*P<*.05).  No significant differences in other lung function indicators. | •None withdrew  •The scores of respiratory exercise compliance were 48.3/44.8 |
| Liu and Pan [28] | •Stigma score (CLCSS^i^)  •Fatigue (Piper Fatigue Scale)  •Peak expiratory flow rate by peak flow meter test  •6-MWD  •Exercise self-efficacy (SEE-C^j^)  •Expectorant volume by microelectronic scales | •Before and aftercare (T0 and T1)  •Expectorant volume on postoperative days 1, 2 and 3 | •The IG had lower CLCSS scores, higher SEE-C scores, a higher proportion of light fatigue, and less proportion of severe fatigue compared with CG (*P<*.05) after the intervention. Expectorant volumes in each time point showed significant differences between the 2 groups. The peak expiratory flow and 6-MWD were better in the IG than in the CG (*P<*.05). | •None withdrew |
| Sun et al [33] | •QOL (Functional Assessment of Cancer Therapy tool)  •Symptoms (MD Anderson Symptom Inventory, Pulmonary Symptom Index)  •Self-efficacy (Self-Efficacy Scale)  •Patient knowledge  •Patient’s activation  •Family caregiver QOL (The City of Hope-Quality of Life)  •Caregiver burden (The Montgomery Borgata Caregiver Burden Scale)  •Family caregiver knowledge  •IG satisfaction | •Predischarge (T0)  •3-7 days  before surgery (T1)  •Days 2 and 7 postdischarge (T2 and T3)  •First postoperative clinic visit (2-4 weeks after discharge) | •Multimedia care statistically improved patients’ emotional dimension scores (*P*=.001), self-efficacy, and surgery-related knowledge (*P<*.05) compared with the CG. Additionally, it enhanced family caregivers’ surgery-related knowledge (*P<*.05). | •Consent rate for screened IG patients: 21 (70%)  •Individuals who withdrew (patient/caregivers): 6/7  •Acceptability rate (patient/caregivers): 3.6-3.7/3.1-3.6  •Usability rate (patient/caregivers): 4.0/4.0  •Satisfaction: 26 (87%) |
| Kadiri et al [15] | •Days from screening to first rehabilitation session  •Days from rehabilitation to surgery date  •Rehabilitation sessions before surgery  •Days of rehabilitation after surgery  •Length of stay  •PPCs  •Intensive therapy unit admission rate  •Readmission within 30 days after surgery  •QOL: European Organization for Research and Treatment of Cancer Core Quality of Life Questionnaire | •Baseline (T0)  •Preoperative (T1)  •Discharge (T2)  •6 weeks after surgery (T3)  •5 months after surgery (T4) | •Patients using the app waited less time and attended more Pulmonary rehabilitation sessions before surgery compared with the CG, improving incremental shuttle walk test distance by a mean of 99 (SD 83) m (*P*<.05) before surgery. | •Withdrawal rate: 10/27 (32%/79%) |
| Finley et al [16] | •Adherence in the last 3 weeks before surgery  •Potential relation between MVPA^k^  •6-MWD | •Enrollment (T0)  •On surgery morning (T1)  •2 weeks after surgery (T2)  •16 weeks after surgery (T3) | •Compared with the baseline, participants exhibited a mean of 20.4 minutes of MVPA per day during the preoperative period. An average of 16.4% of participants achieved the 30-minute MVPA target before surgery, with an average improvement of 13.8 m on 6-MWD. | •Preoperative activities completion rate: 14 (79%)  •Rate of successful device synchronization: 13 (71%) |
| Yang et al [29] | •QOL: QLQ C30 + European Organization for Research and Treatment of Cancer Quality of Life Questionnaire-Lung Cancer 13 (QLQ-LC13)  •Muscular strength  •2-minute walk test  •Patient’s satisfaction: Patient Global Assessment | •Baseline (T0)  •Week 6 (T1)  •Week 12 (T2) | •Overall satisfaction with the Smart After-Care Program was very high after the intervention, with significant improvements in QOL (*P<*.05), lower limb muscle strength (*P*=.01), and 2-minute walking distance (*P*=.028); 97% of patients completed the study and were satisfied with using the equipment. | •8 withdrawals (16%) |
| Qin et al [26] | •6-MWD  •Knowledge, Attitude/Belief, and Practice of Pulmonary rehabilitation  •Duration of chest drainage  •Length of stay after surgery  •Patient’s satisfaction: PGA | •At admission(T0)  •At discharge  (T1) | •The score of the Knowledge, Attitude/Belief, Practice of Pulmonary rehabilitation, 6MWD, and PGA score in the IG was statistically better than that in the CG (*P<*.05). The chest tube duration in the IG was lower than that in the CG (*P*=.03). | •None withdrew |
| Chen et al [34] | •Lung Function: FVC^l^, FEV_1_^m^, FVC/FEV_1_%, FEV_1_%  •QOL: QLQ-C30  •Self-rating depression scale, Self-Rating Anxiety Scale  •6-MWD  •Chronic obstructive pulmonary disease assessment test  •Postoperative oxygen therapy  •Chest tube duration  •Postoperative hospitalization | •At admission (T0)  •After intervention (T1) | •The incidence of postoperative atelectasis, pulmonary infection, hypoxemia, postoperative oxygen therapy time, chest tube duration, and postoperative hospital stay in the IG was statistically lower than those in the CG (*P<*.05); FEV_1_, FVC, and FVE_1_% of the IG were statistically higher than those of the CG after the intervention (all *P*<.05). | •None withdrew |

^a^IG: intervention group.

^b^CG: control group.

^c^QOL: quality of life.

^d^6-MWD: 6-minute walking test.

^e^mMRC: modified Medical Research Council.

^f^HADS-A: Hospital Anxiety and Depression Scale-Anxiety.

^g^HADS-D: Hospital Anxiety and Depression Scale-Depression.

^h^PPC: postoperative pulmonary complication.

^i^CLCSS: Chinese Lung Cancer Patient Stigma Scale.

^j^SEE-C: Chinese version of the Exercise Self-Efficacy Scale.

^k^MVPA: moderate to vigorous physical activity.

^l^FVC: forced vital capacity.

^m^FEV_1_: forced expiratory volume in 1 second.
